# WDR23 prevents bone loss by promoting autophagic degradation of TRAF6 in osteoclastogenesis

**DOI:** 10.1038/s41413-026-00560-2

**Published:** 2026-09-10

**Authors:** Hye-Won Park, Jungeun Yu, Jiyeon Yu, Jinseon You, Yeon Hee Kook, Joo-Yong Lee, Taesoo Kim, Chul-Ho Lee, Hee-Chung Chung, Jong-Soon Choi, Sangkyu Lee, Jaerang Rho

**Affiliations:** 1 Department of Microbiology and Molecular Biology, Chungnam National University, Daejeon, South Korea; 2 Center for Cognition and Sociality, Institute for Basic Science (IBS), Daejeon, South Korea; 3 Graduate School of Analytical Science and Technology, Chungnam National University, Daejeon, South Korea; 4 Herbal Medicine Research Division, Korea Institute of Oriental Medicine (KIOM), Daejeon, South Korea; 5 Laboratory Animal Center, Korea Research Institute of Bioscience & Biotechnology (KRIBB), Daejeon, South Korea; 6 Research Center for Digital Omics, Korea Basic Science Institute (KBSI), Daejeon, South Korea

**Keywords:** Homeostasis, Bone

## Abstract

Tumor necrosis factor receptor-associated factor 6 (TRAF6) is a pivotal adaptor molecule in the receptor activator of nuclear factor-κB (RANK) and its ligand (RANKL) signaling pathways, which are essential for osteoclastogenesis. In this study, we identified WD40 repeat-containing protein 23 (WDR23), also known as DDB1-CUL4 associated factor 11 (DCAF11), as a novel binding partner of TRAF6. Our findings demonstrate that WDR23/DCAF11 acts as a negative feedback regulator of RANK/RANKL-induced osteoclastogenesis by promoting the autophagy-dependent degradation of TRAF6. Notably, RANKL induced the upregulation of WDR23 expression during osteoclastogenesis. WDR23 physically interacted with the TRAF domain of TRAF6 via the WD40 repeat domains 1 and 2 of WDR23, resulting in reduced TRAF6 protein stability by its autophagy-dependent degradation during osteoclastogenesis. By modulating TRAF6 protein levels, WDR23 attenuated RANKL signaling cascades, including nuclear factor-κB and mitogen-activated protein kinases, thereby downregulating the expression of osteoclastogenic markers, such as nuclear factor of activated T-cell c1, tartrate-resistant acid phosphatase, dendritic cell-specific transmembrane protein, V-ATPase subunit d2 and cathepsin K. Conversely, WDR23 knockdown or deficiency enhanced RANKL-induced osteoclastogenesis by preventing the autophagy-dependent degradation of TRAF6. WDR23-deficient mice exhibit an osteoporotic bone phenotype characterized by elevated osteoclast formation and reduced bone mass. Collectively, these results establish WDR23 as a key negative feedback regulator of RANKL-induced osteoclastogenesis via autophagy-mediated TRAF6 degradation and underscore its potential as a therapeutic target for bone disorders associated with aberrant osteoclast formation and function.

**Figure:**
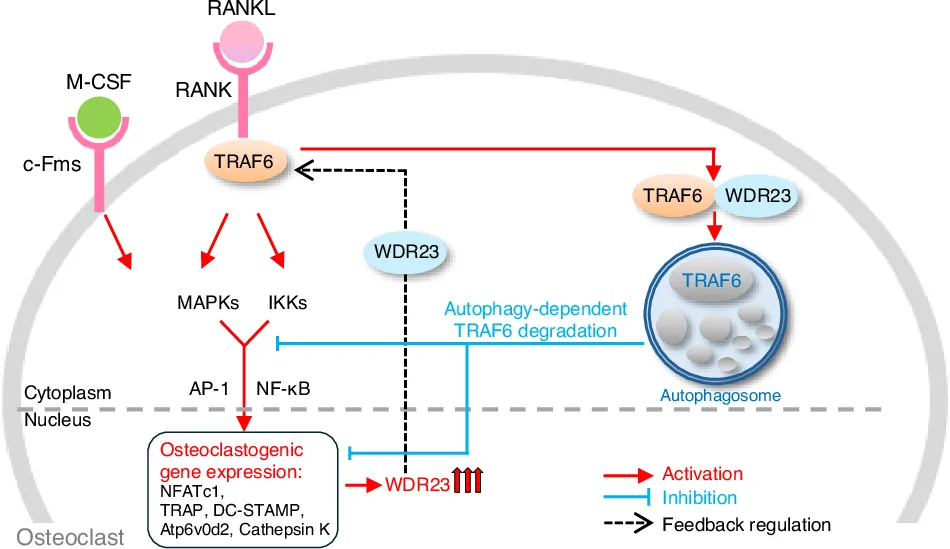

## Introduction

Bone homeostasis is tightly regulated by the dynamic balance between bone-forming osteoblasts (OBs) and bone-resorbing osteoclasts (OCs).^1^ Dysregulation of OC formation and function is linked to skeletal disorders, including osteoporosis, rheumatoid arthritis and Paget’s disease.^1–3^ OCs are large, multinucleated cells that arise from monocyte/macrophage lineage precursors.^3^ The differentiation and maturation of OCs, collectively referred to as osteoclastogenesis, are driven primarily by two key cytokines: macrophage colony-stimulating factor (M-CSF) and receptor activator of nuclear factor-κB (RANK) ligand (RANKL), both of which are produced by OBs and immune cells.^1–3^

Tumor necrosis factor receptor (TNFR)-associated factor 6 (TRAF6) is essential for immune responses, development and homeostasis, mediating signaling pathways from the TNFR and toll-like receptor (TLR)/interleukin-1 receptor (IL-1R) families.^4^ The activation of TRAF6 through RANK/RANKL signaling is crucial for osteoclastogenesis.^2,3,5^ Upon RANKL stimulation, TRAF6 is recruited to the cytoplasmic tail of RANK, initiating downstream signaling cascades that involve adaptors and kinases, such as nuclear factor κB (NF-κB) essential modulator, inhibitor of κB (IκB), IκB kinases (IKKs), Akt and mitogen-activated protein kinases (MAPKs), including p38, c-Jun N-terminal kinase (JNK) and extracellular signal-regulated kinase (ERK).^2,3,5^ These signaling cascades subsequently activate transcription factors, such as NF-κB and activator protein-1 (AP-1), leading to the induction of nuclear factor of activated T-cell c1 (NFATc1), the master transcriptional regulator of osteoclastogenesis.^3^ NFATc1 promotes OC formation and maturation by upregulating osteoclastogenic markers, such as tartrate-resistant acid phosphatase (TRAP), dendritic cell-specific transmembrane protein (DC-STAMP), v-ATPase subunit d2 (Atp6v0d2) and cathepsin K.^3,5–7^ The critical role of TRAF6 in osteoclastogenesis is highlighted by severe osteopetrosis observed in mice deficient in TRAF6, RANK or RANKL due to impaired OC formation.^8–10^

The stability and turnover of TRAF6 are crucial for regulating the TNFR and TLR/IL-1R signaling pathways.^11–15^ Structurally, TRAF6 contains an N-terminal RING domain and zinc finger motif, a central coiled-coil domain and a highly conserved C-terminal TRAF domain.^16,17^ The RING domain of TRAF6 has E3 ubiquitin ligase activity, catalyzing the attachment of ubiquitin molecules to itself (autoubiquitination) or to downstream signaling proteins.^18^ Ubiquitination of TRAF6 plays a pivotal role in regulating signaling pathways.^19^ Specifically, K^63^-linked ubiquitination promotes signaling pathways by recruiting and activating downstream adaptors/kinases, including NF-κB essential modulator, IκB, Akt and MAPKs, whereas K^48^-linked ubiquitination targets signaling molecules for ubiquitin-proteasomal degradation, thereby attenuating the signaling pathway.^11,18,19^ TRAF6 stability and ubiquitin ligase activity are further regulated by interactions with cellular proteins, enabling precise control over its role in various cellular processes, including osteoclastogenesis.^4^ Autophagy, a conserved cellular degradation process, is crucial for OC formation and function.^20^ TRAF6 stability and turnover in signaling pathways are also modulated by autophagy-dependent degradation.^21–25^ Notably, TRAF6 E3 ubiquitin ligase activity is implicated in the initiation of autophagy.^26–28^ Therefore, the regulation of TRAF6 through ubiquitin–proteasomal and autophagy-dependent pathways is crucial for maintaining the balance of OC formation and function.

WD40 repeat-containing protein 23 (WDR23), also known as DDB1-CUL4 associated factor 11 (DCAF11), is a member of the family of WD40 repeat proteins.^29,30^ WD40 repeat proteins play crucial roles in diverse biological processes, including signaling, transcriptional regulation, cell division, cytoskeleton assembly, apoptosis and autophagy.^31,32^ The WD40 repeat domain is a highly conserved structural motif composed of approximately 40–60 amino acids, characterized by a conserved Gly-His (GH) sequence near its N-terminus and a conserved Trp-Asp (WD) sequence at its C-terminus.^31,32^ WDR23, which contains seven WD40 repeat domains, has been identified to be associated with the CUL4A-DDB1 E3 ubiquitin ligase complex.^29,30,33^ In this complex, WDR23 regulates the stability of nuclear factor erythroid 2-related factor (NRF2) and SKN-1 (a functional equivalent NRF2 in *Caenorhabditis elegans*) through ubiquitin-dependent proteasomal degradation, which is essential for the cellular stress response and longevity.^30,34^ Furthermore, the CUL4-DDB1-WDR23 E3 ubiquitin ligase complex is involved in the regulation of the cell cycle, cell division and telomere length by promoting ubiquitin-dependent proteasomal degradation of key cellular mediators, including p21, stem‒loop binding protein, centromere protein A and KRAB-associated protein 1.^35–38^ While the specific role of WDR23 in autophagy-dependent protein degradation processes remains unknown, other members of the WDR family are known to participate in the regulation of autophagy.^39–41^ This connection indicates the potential functional relevance of WDR23 in the autophagy-dependent protein degradation pathway.

In this study, we identified WDR23 as a novel binding partner of TRAF6. Our findings revealed that WDR23 is upregulated in response to RANKL stimulation and functions as a negative regulator of osteoclastogenesis by promoting the autophagy-dependent degradation of TRAF6. WDR23-deficient (*WDR23*^*−*/*−*^) mice exhibited enhanced OC formation and reduced bone mass. These findings suggest that WDR23 serves as a negative feedback regulator in RANKL-induced osteoclastogenesis, offering new insights into the molecular mechanisms underlying bone homeostasis.

## Results

### WDR23 is a novel TRAF6-binding partner

**Fig. 1:**
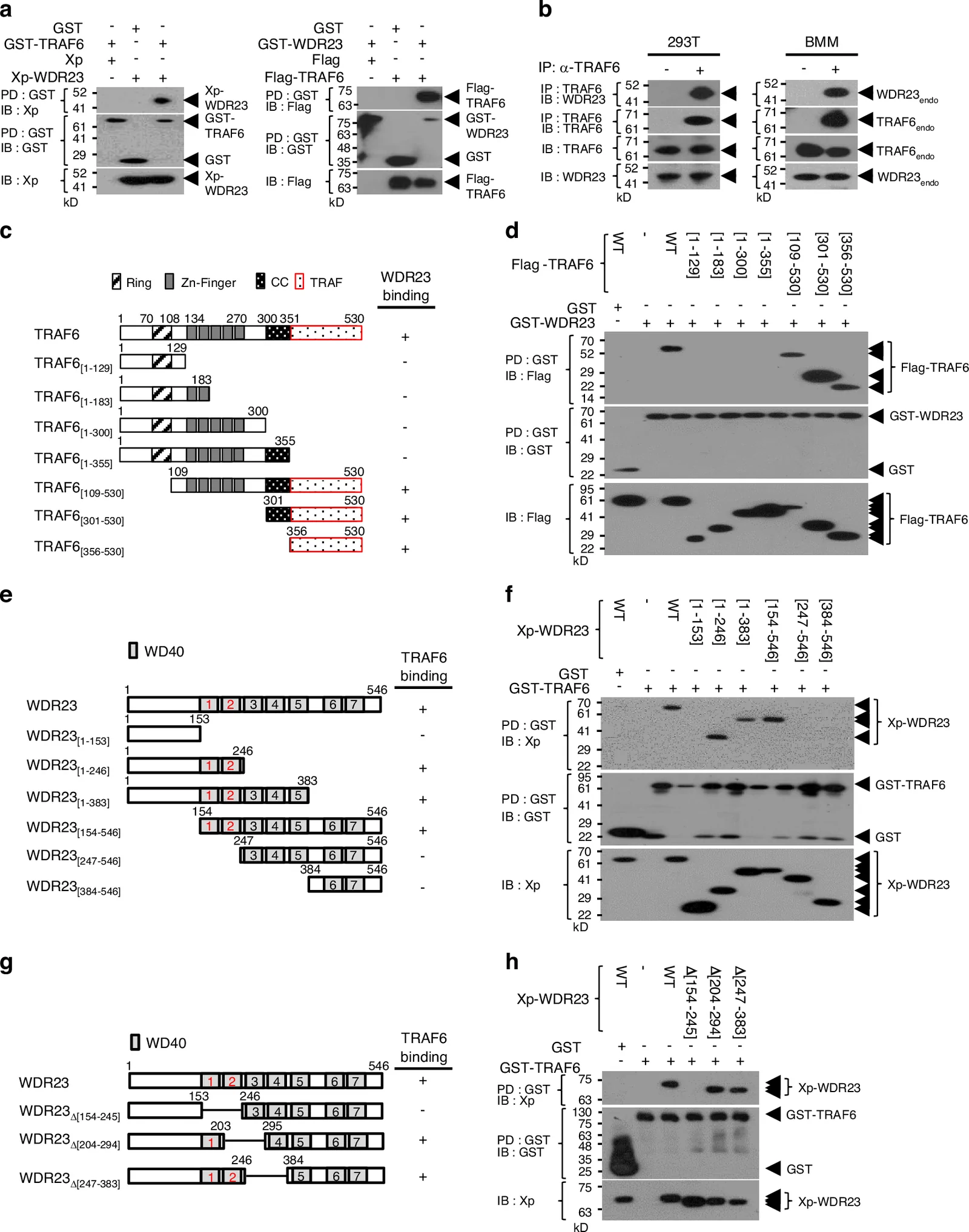
TRAF6 interacts with WDR23. **a** GST-TRAF6 interacts with Xp-WDR23 in 293T cells (left panels). GST-TRAF6 was pulled down (PD) using GST beads, and the bound proteins were detected by immunoblotting (IB) with anti-Xp. GST alone was used as a control. GST-WDR23 interacts with Flag-TRAF6 in 293T cells (right panels). **b** Endogenous TRAF6 interacts with WDR23 in 293T cells (left panels). Endogenous TRAF6 (TRAF6_endo_) was immunoprecipitated (IP) with anti-(α-)TRAF6 antibodies, and proteins bound to TRAF6 were visualized by IB with anti-WDR23 antibodies (WDR23_endo_). Endogenous TRAF6 interacts with WDR23 in BMMs (right panels). **c** Schematic diagram of TRAF6 and its deletion mutants. The domains of TRAF6, including the RING finger domain (RING), zinc finger domain (Zn-Finger), coiled-coil (CC) region and TRAF domain, are shown with corresponding amino acid positions. The interaction of TRAF6-WDR23 are summarized on the right. **d** The TRAF domain of TRAF6 interacts with WDR23. **e**, **g** Schematic diagram of WDR23 and its deletion mutants. The seven WD40 repeat domains are numbered 1-7 within a gray box. **f**, **h** WD40 repeat domains 1 and 2 of WDR23 interact with TRAF6

To identify novel protein partners of TRAF6, we conducted proteomic analyses using 293T cells expressing Flag-TRAF6.^17^ Following immunoprecipitation (IP) with anti-Flag beads, several TRAF6-bound protein candidates, approximately 68 kD in size, were excised and analyzed using nanoscale liquid chromatography coupled with tandem mass spectrometry (Fig. S1A). Among these candidates, WDR23 was identified as a potential binding partner of TRAF6 by immunoblotting (IB) with an anti-WDR23 antibody (Fig. S1B). Using a GST-tagged expression system in 293T cells, the interaction between TRAF6 and WDR23 was further validated by GST bead pull-down (PD) assays (Fig. 1a). Furthermore, in a GST-PD assay, WDR23 was observed to interact with other TRAF family members recruited to RANK (Fig. S1C). To confirm the interaction between TRAF6 and WDR23 at the endogenous protein level, a TRAF6 IP assay was conducted. The interaction between endogenous TRAF6 and WDR23 was detected in 293T cells (Fig. 1b, left) and bone marrow-derived macrophages (BMMs) (Fig. 1b, right). Upon RANKL stimulation, the association between endogenous TRAF6 and WDR23 was also observed in bone marrow-derived OCs (BMOCs) (Fig. S1D). Next, we examined whether WDR23 is recruited to the RANK signaling complex. An anti-Flag IP assay revealed that WDR23 associates with Flag-RANK via TRAF6 but not with the Flag-RANK-AAA mutant lacking three TRAF6-binding consensus sites (T6BSs) (Fig. S2A). Similarly, a GST-PD assay using GST-RANK_cyto_ (GST-fused RANK cytoplasmic tail) further confirmed that WDR23 was recruited to GST-RANK_cyto_ via TRAF6 but not to the GST-RANK_cyto_-AAA mutant (Fig. S2B).

To identify the TRAF6 region required for the WDR23 interaction, we utilized TRAF6 deletion mutants lacking the N-terminal and C-terminal regions (Fig. 1c). WDR23 interacted with TRAF6_[109-530]_, TRAF6_[301-530]_ and TRAF6_[356-530]_, indicating that the TRAF domain between residues 356 and 530 is required for the interaction (Fig. 1d). Functional rescue experiments revealed that the impaired OC formation in TRAF6^−/−^ macrophages was not restored by the expression of either WDR23-binding TRAF6 mutant Flag-TRAF6_[1-355]_ or WDR23-nonbinding TRAF6 mutant Flag-TRAF6_[356-530]_, whereas expression of full-length TRAF6 effectively rescued OC differentiation (Fig. S2C). These results indicate that TRAF6 deletion mutants lacking either the N-terminal or C-terminal region are insufficient to support OC differentiation, despite the requirement of the C-terminal TRAF domain for WDR23 binding. To explore the specific domain of WDR23 that interacts with TRAF6, we generated WDR23 deletion mutants targeting the N-terminal and C-terminal regions (Fig. 1e). GST-PD assays revealed that TRAF6 bound to the WDR23_[1-246]_, WDR23_[1-383]_ and WDR23_[154-546]_ mutants but not to the WDR23_[1-153]_, WDR23_[247-546]_ or WDR23_[384-546]_ mutants (Fig. 1f). These results indicate that the region from residues 154 to 246, containing WD40 repeat domains 1 and 2, is critical for the interaction. Furthermore, GST-PD assays using WDR23 internal deletion mutants revealed that TRAF6 interacted with WDR23_Δ[204-294]_ and WDR23_Δ[247-383]_ but not with WDR23_Δ[154-245]_, confirming the importance of the WD40 repeat domains 1 and 2 (Fig. 1g, h). To precisely define the specific domain of WDR23 that interacts with TRAF6, single WD40 repeat domain deletion mutants of WDR23 were generated. However, all single WD40 repeat domain deletion mutants retained the ability to interact with TRAF6 (Fig. S3). Taken together, these results indicate that the TRAF domain of TRAF6 is required for its interaction with WD40 repeat domains 1 and 2 of WDR23.

### WDR23 acts as a negative feedback regulator of RANKL-induced osteoclastogenesis

**Fig. 2:**
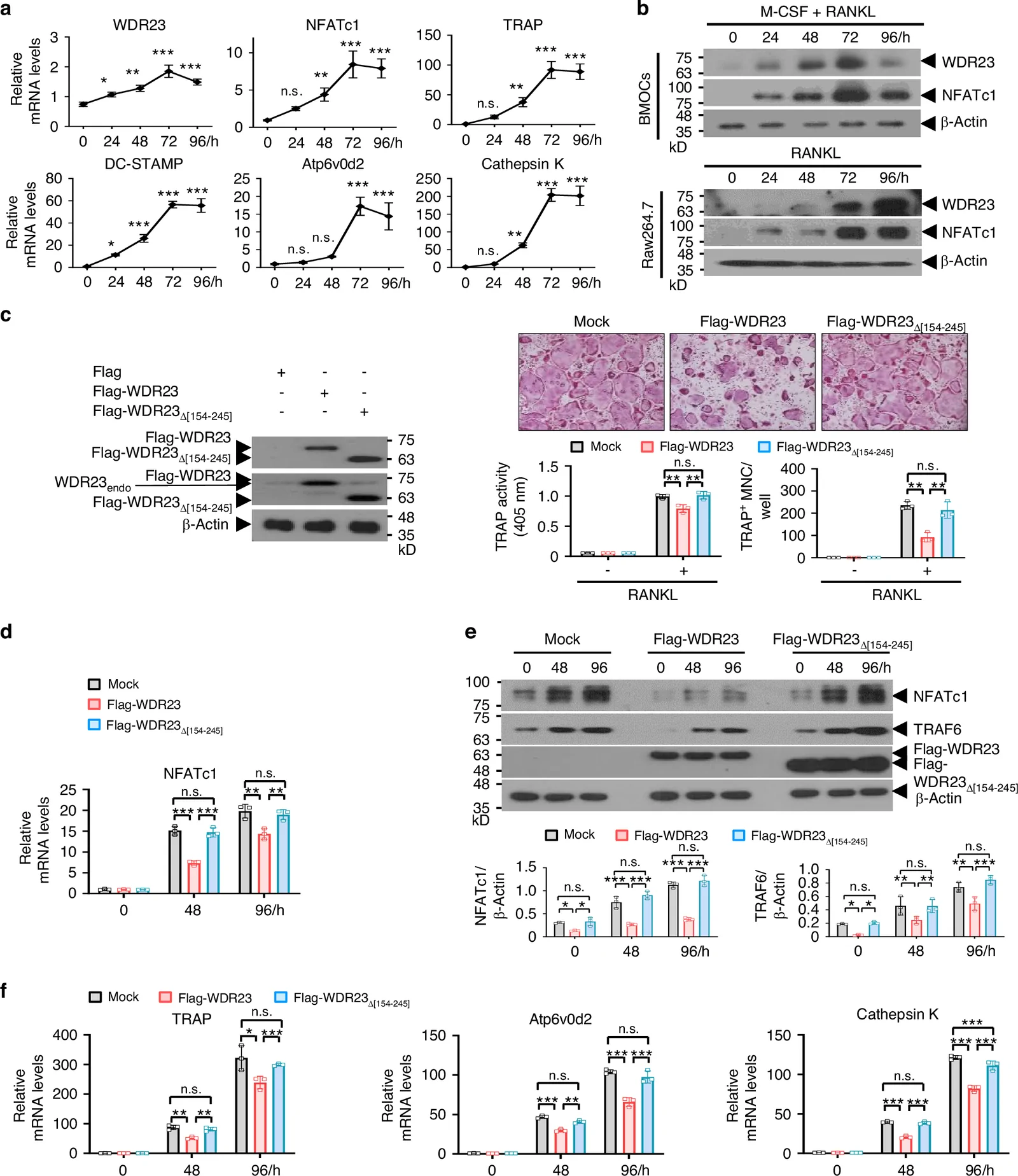
WDR23 induced by M-CSF and RANKL stimulation is negatively involved in osteoclastogenesis. **a** Real-time PCR analysis of WDR23 induction. The gene expression levels of osteoclastogenic markers in BMOCs were quantified by real-time PCR. β-Actin was used as the control. **b** Immunoblotting analysis of WDR23 induction in BMOCs and RAW 264.7 cells. **c** Inhibition of osteoclastogenesis by forced expression of WDR23. BMMs expressing Flag-WDR23 or Flag-WDR23_Δ[154-245]_ were differentiated into BMOCs. The expression of Flag-WDR23 was analyzed by immunoblotting with anti-Flag and anti-WDR23 antibodies (WDR23_endo_). Osteoclastogenesis was assessed by TRAP staining (original magnification ×50), and quantified by a TRAP activity assay and by counting TRAP-positive multinucleated OCs (TRAP^+^ MNCs (≥three nuclei)). An empty vector (Flag) was used as the control. Downregulation of NFATc1 expression by WDR23 overexpression, as determined by real-time PCR (**d**) and immunoblotting (**e**). The relative levels of NFATc1 and TRAF6 were normalized to β-actin (**e**). **f** Downregulation of osteoclastogenic markers by forced expression of WDR23. The data are shown as the means ± SD. ^*^P < 0.05. ^**^P < 0.01. ^***^P < 0.001. n.s., not significant

To investigate the role of WDR23 in osteoclastogenesis, we first examined the regulation of WDR23 expression during osteoclastogenesis. BMMs were differentiated into BMOCs by stimulation with RANKL (200 ng/mL) in the presence of M-CSF (50 ng/mL) for 4 d. Quantitative real-time PCR analysis revealed a progressive increase in WDR23 expression (1.4 to 2.5-fold), peaking on Day 3 and slightly decreasing by Day 4 (Fig. 2a). However, the induction of WDR23 expression was notably lower than the expression of OC markers, including NFATc1, TRAP, DC-STAMP, Atp6v0d2 and cathepsin K (Fig. 2a). Consistently, immunoblotting analysis further confirmed the upregulation of WDR23 expression in BMMs following stimulation with RANKL and M-CSF, as well as in RAW 264.7 cells upon RANKL stimulation, with peak expression observed on Day 4 in RAW 264.7 cells (Fig. 2b). These results indicate that WDR23 expression is induced by RANKL stimulation during osteoclastogenesis. To elucidate the role of the TRAF6-WDR23 interaction, we investigated its impact on RANKL-induced osteoclastogenesis. BMMs were transduced with retroviral vectors to overexpress Flag-WDR23 or a TRAF6-nonbinding mutant, Flag-WDR23_Δ[154-245]_, followed by puromycin selection (Fig. 2c, left). Puromycin-resistant BMMs were subsequently differentiated into BMOCs with RANKL and M-CSF stimulation (Fig. 2c, right top). Compared with the mock control, forced expression of Flag-WDR23 significantly inhibited both TRAP activity (0.79 ± 0.06 vs. 0.99 ± 0.04) and the number of TRAP^+^ multinucleated OCs (92.3 ± 21.5 vs. 236 ± 17.5), whereas no inhibitory effects were observed with the Flag-WDR23_Δ[154-245]_ mutant (Fig. 2c, right bottom). Furthermore, forced expression of Flag-WDR23 significantly reduced NFATc1 mRNA and protein levels, whereas the TRAF6-nonbinding mutant had no such effect (Fig. 2d, e). Consistently, the expression of NFATc1-downstream OC markers, including TRAP, Atp6v0d2 and cathepsin K, was suppressed by Flag-WDR23 expression but not by Flag-WDR23_Δ[154-245]_ expression (Fig. 2f). Collectively, these results indicate that WDR23 acts as a negative feedback regulator of osteoclastogenesis via its interaction with TRAF6.

TRAF6-mediated activation of NF-κB and MAPKs within the RANKL signaling pathway is crucial for OC differentiation and maturation through the induction of NFATc1 expression.^2,3^ To determine whether WDR23 regulates RANKL-induced signaling, we analyzed the effects of WDR23 on this pathway. BMMs transduced with Flag-WDR23 using a retroviral gene transfer system and selected with puromycin were stimulated with 200 ng/mL RANKL for the indicated durations. The activation of NF-κB and MAPKs following RANKL stimulation was analyzed by immunoblotting and a luciferase reporter assay (Fig. S4). Compared with mock treatment, forced expression of WDR23 resulted in a reduction in the phosphorylation of IκBα and p65, which are markers of NF-κB activation, as well as a decrease in the phosphorylation of MAPKs, including p38, JNK and ERK (Fig. S4A). Consistent with these observations, a reporter assay revealed that WDR23 expression inhibited TRAF6-mediated transcriptional activation of both NF-κB and AP-1 in a dose-dependent manner (Fig. S4B, C). Collectively, these findings indicate that WDR23 negatively regulates RANKL-induced signaling by modulating the TRAF6-mediated signaling pathway.

### WDR23 knockdown enhances RANKL-induced osteoclastogenesis

**Fig. 3:**
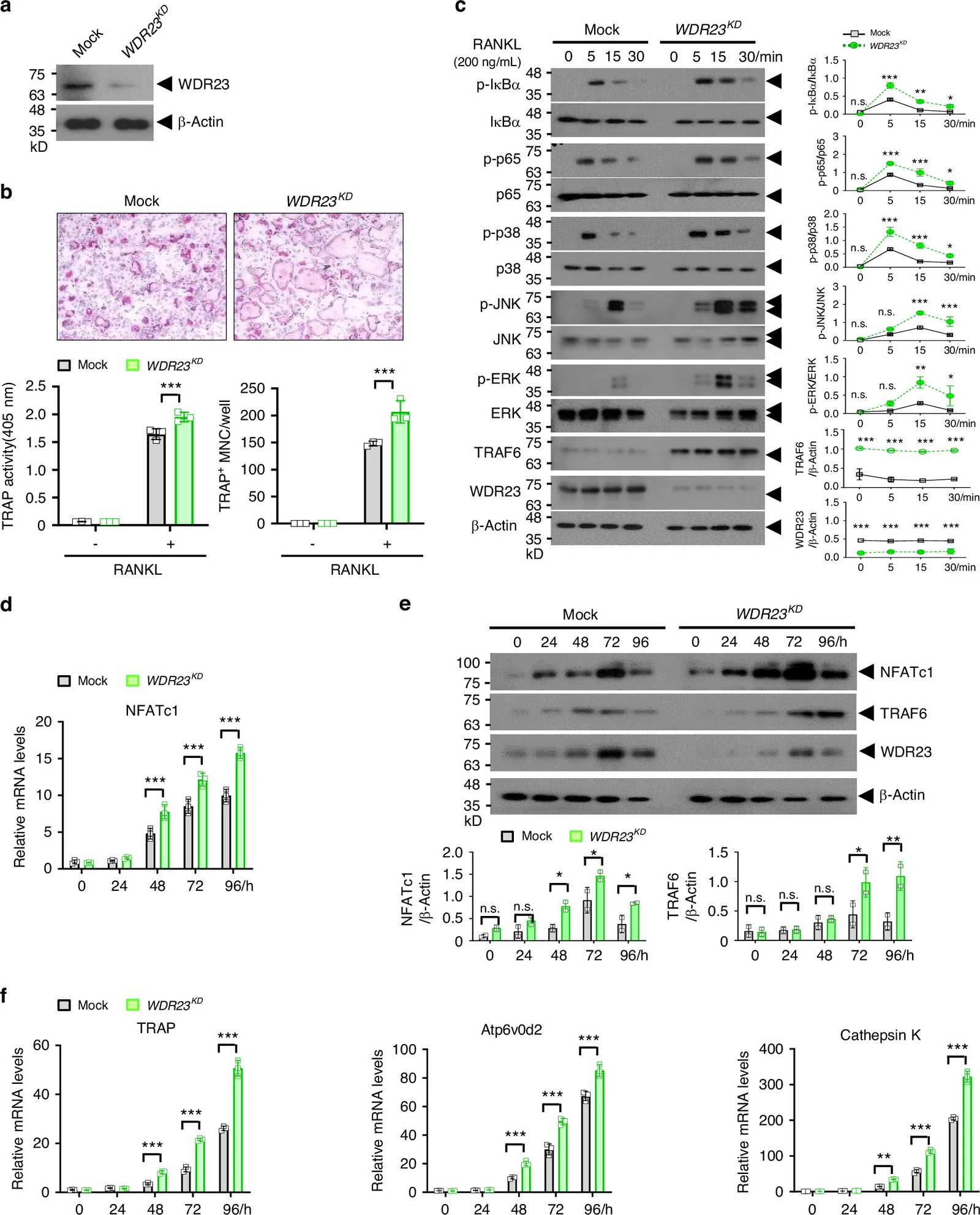
Knockdown of WDR23 enhances osteoclastogenesis. **a** Verification of WDR23 knockdown (*WDR23*^*KD*^) in BMMs. Mock, GFP^KD^ (empty) vector control. **b** Increased osteoclastogenesis in *WDR23*^*KD*^-BMMs. Osteoclastogenesis was assessed by TRAP staining (original magnification ×50) and quantified by a TRAP activity assay and by counting TRAP-positive multinucleated OCs (TRAP^+^ MNCs (≥three nuclei)). **c** Upregulation of RANKL-induced signaling in *WDR23*^*KD*^-BMMs. The RANKL-induced signaling pathway was subsequently analyzed by immunoblotting with antibodies that recognize phosphorylated and total IκBα, NF-κB p65, p38, JNK and ERK. The relative levels of the phosphorylated forms were calculated after normalization to the total protein input. The relative levels of WDR23 and TRAF6 were normalized with β-actin. Increased NFATc1 expression in *WDR23*^*KD*^-BMMs, as determined by real-time PCR (**d**) and immunoblotting (**e**). **f** Upregulation of osteoclastogenic markers following *WDR23*^*KD*^. The data are shown as the means ± SD. ^*^P < 0.05. ^**^P < 0.01. ^***^P < 0.001. n.s., not significant

To further investigate the impact of WDR23 expression on osteoclastogenesis, we generated WDR23-knockdown (*WDR23*^*KD*^) BMMs using a retroviral gene transfer system followed by puromycin selection, confirming the effective knockdown of WDR23 expression (Fig. 3a). In RANKL-induced osteoclastogenesis, *WDR23*^*KD*^ led to a significant increase in both TRAP activity (1.95 ± 0.09 vs. 1.64 ± 0.1) and the number of TRAP^+^ multinucleated OCs (207.0 ± 20.8 vs. 148.0 ± 4.4) compared to the mock control (Fig. 3b). Furthermore, the activation of the RANKL-induced NF-κB and MAPK pathways was markedly greater in *WDR23*^*KD*^ BMMs compared to the mock control (Fig. 3c). Consistently, the mRNA and protein levels of NFATc1 were significantly elevated in *WDR23*^*KD*^ BMOCs (Fig. 3d, e). Consequently, the expression of NFATc1 downstream OC markers, including TRAP, Atp6v0d2 and cathepsin K, was upregulated following *WDR23*^*KD*^ (Fig. 3f). These results suggest that *WDR23*^*KD*^ enhances RANKL-induced osteoclastogenesis by promoting the activation of key signaling pathways and upregulating the expression of OC-specific markers.

### WDR23 promotes TRAF6 degradation via an autophagosomal pathway

Having observed a dose-dependent decrease in TRAF6 protein levels following WDR23 expression (Figs. 2 and S4), we further investigated the impact of WDR23 on TRAF6 protein stability. In 293T cells, the expression of WDR23 resulted in a dose-dependent reduction in both exogenous and endogenous TRAF6 levels (Fig. S5A, B). Deletion mutant assays revealed that WDR23 expression reduced the stability of the WDR23-binding Flag-TRAF6_[356-530]_ mutant (Fig. S5G, I), whereas the stability of WDR23-nonbinding TRAF6 mutants (Flag-TRAF6_[1-129]_, Flag-TRAF6_[1-183],_ Flag-TRAF6_[1-300]_ and Flag-TRAF6_[1-355]_) remained unaffected (Fig. S5C–F, H). A chase experiment using cycloheximide (CHX), a de novo protein synthesis inhibitor, revealed that WDR23 significantly reduced TRAF6 stability in a time-dependent manner compared with that in the control condition (Flag-TRAF6 alone) (Fig. S5J). Consistently, WDR23 selectively reduced the stability of the WDR23-binding Flag-TRAF6_[356-530]_ mutant (Fig. S5N), but not that of the WDR23-nonbinding TRAF6 mutants (Fig. S5K–M). To exclude transcriptional regulation as a factor influencing TRAF6 protein levels, the CMV promoter was replaced with the EF1α promoter for Flag-TRAF6 expression. The reduction in TRAF6 stability caused by WDR23 overexpression was unaffected by changes in promoter activity (Fig. S6). Given that WDR23 interacts with all TRAF family members (Fig. S1C), the stability of other TRAF proteins was examined following WDR23 expression. Notably, among the TRAF family members, only TRAF6 stability was reduced by WDR23 expression (Fig. S7). These findings indicate that WDR23 selectively regulates TRAF6 stability. To directly evaluate the effect of the TRAF6-WDR23 interaction on TRAF6 stability, we utilized a TRAF6-nonbinding mutant, Flag-WDR23_Δ[154-245]_. In 293T cells, the expression of Xp-WDR23 reduced Flag-TRAF6 protein levels in a dose-dependent manner, whereas the Xp-WDR23_Δ[154-245]_ mutant had no effect (Fig. S5O). Consistent with these results, immunofluorescence analysis revealed that the expression of Flag-WDR23, but not the TRAF6-nonbinding mutant Flag-WDR23_Δ[154-245]_, significantly reduced the levels of TRAF6 colocalized with WDR23 in a time-dependent manner in both MEFs and BMMs (Fig. S8). Compared with that of the mock control, endogenous TRAF6 stability in BMOCs was significantly reduced by WDR23 expression but was unaffected by the Xp-WDR23_Δ[154-245]_ mutant (Fig. 2). Conversely, TRAF6 stability was greater in *WDR23*^*KD*^ BMOCs compared to the mock control (Fig. 3). To confirm that WDR23 does not regulate TRAF6 at the transcriptional level, we measured TRAF6 mRNA levels in BMOCs. The results revealed no significant changes in TRAF6 mRNA levels in either WDR23-expressing or *WDR23*^*KD*^ BMOCs (Fig. S9A, B). Taken together, these results indicate that the TRAF6-WDR23 interaction selectively reduces TRAF6 protein stability independent of transcriptional regulation.

**Fig. 4:**
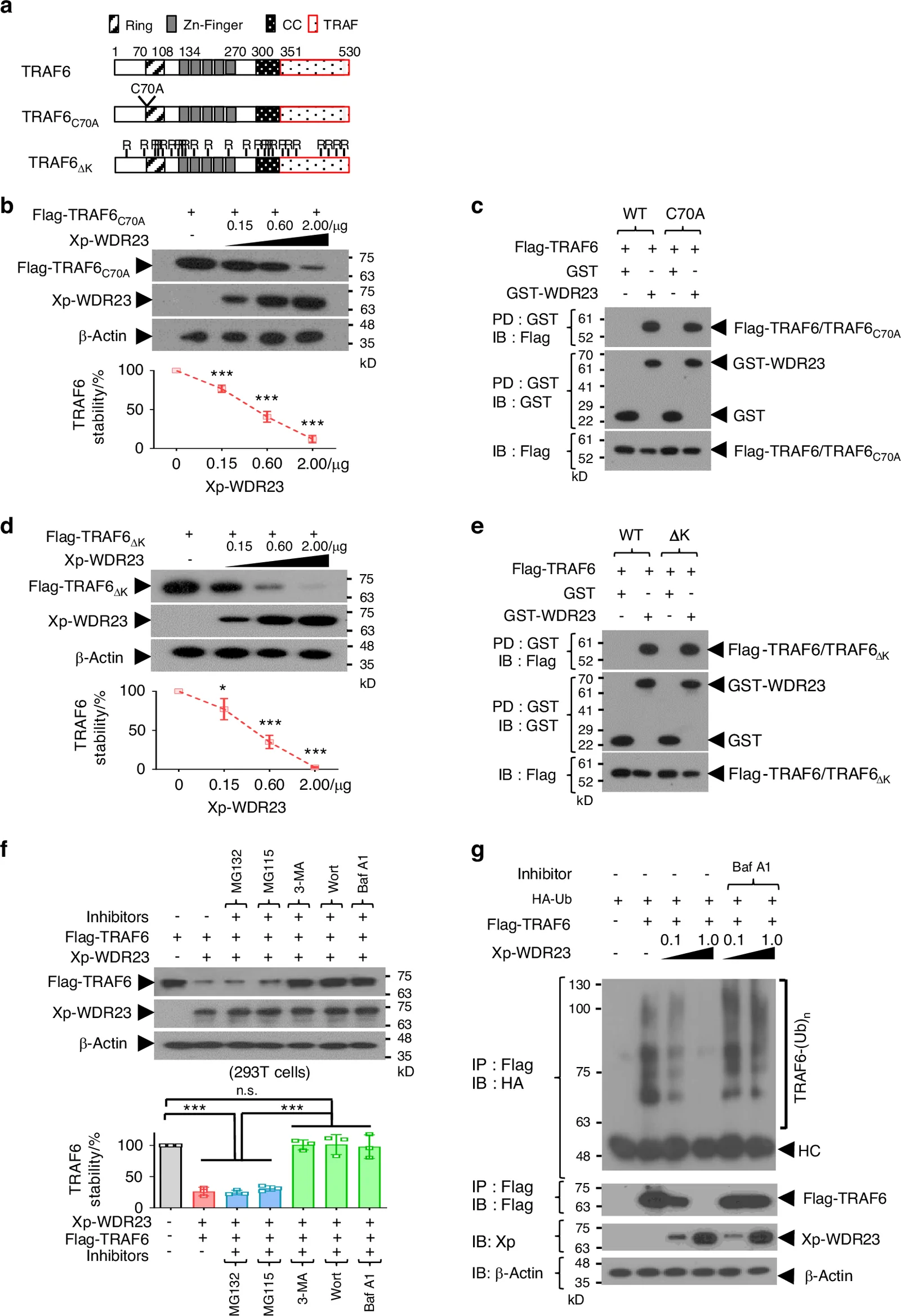
WDR23 expression promotes autophagy-dependent TRAF6 degradation rather than via the ubiquitin–proteasome pathway. **a** Schematic diagram of TRAF6 mutants. The TRAF6_C70A_ mutation (C70A: substitution of cysteine (C) at position 70 in the RING domain with alanine (A)) and the Lys-deficient TRAF6 mutation (TRAF6_∆K_; all lysine (K) residues in TRAF6 mutated to arginine (R)) are indicated above the diagram. **b** WDR23 reduces TRAF6_C70A_ levels in 293T cells. At 24 h post-transfection, cell lysates were immunoblotted, and Flag-TRAF6_C70A_ levels were quantified relative to β-actin. **c** WDR23 interacts with the TRAF6_C70A_ mutant. **d** WDR23 reduces TRAF6_∆K_ levels in 293T cells. **e** WDR23 interacts with the TRAF6_∆K_ mutant. **f** WDR23-mediated TRAF6 degradation is rescued by autophagy inhibitors in 293T cells. Cells were treated for 8 h with ubiquitin–proteasome inhibitors (MG132, 20 μmol/L; MG115, 20 μmol/L) or autophagy inhibitors (3-methyladenine (3-MA), 5 mmol/L; wortmannin (Wort), 5 μmol/L; bafilomycin A1 (Baf A1), 1 μmol/L). **g** WDR23 does not affect TRAF6 ubiquitination activity. The level of TRAF6 ubiquitination (TRAF6-(Ub)_n_) was analyzed by immunoblotting with anti-HA antibodies. The data are shown as the means ± SD. ^*^P < 0.05. ^***^P < 0.001. n.s., not significant

Cellular protein degradation occurs predominantly via two main pathways: the ubiquitin–proteasomal pathway and the autophagosomal pathway.^42^ To investigate the mechanism underlying WDR23-mediated TRAF6 degradation, we examined the role of TRAF6 ubiquitination. Using an E3 ubiquitin ligase-defective TRAF6 mutant (TRAF6_C70A_) and a lysine-deficient mutant (TRAF6_∆K_, where all lysine residues are mutated to arginine), we observed a dose-dependent reduction in Flag-TRAF6_C70A_ protein levels upon WDR23 expression in 293T cells (Fig. 4a, b). Furthermore, the interaction between TRAF6 and WDR23 was not disrupted by the E3 ubiquitin ligase-defective mutation in TRAF6 (Fig. 4c). Similarly, WDR23 effectively promoted the degradation of the lysine-deficient TRAF6_∆K_ mutant, and the TRAF6-WDR23 interaction was unaffected by the lysine-deficient TRAF6_∆K_ mutation (Fig. 4d, e). These findings indicate that neither the E3 ubiquitin ligase activity of TRAF6 nor its ubiquitination is required for WDR23-mediated TRAF6 degradation. To distinguish between the ubiquitin–proteasomal and autophagosomal pathways in WDR23-mediated TRAF6 degradation, we used specific inhibitors. In 293T cells coexpressing Flag-TRAF6 and Xp-WDR23, we treated cells with ubiquitin‒proteasome inhibitors (MG132 and MG115) and autophagosome inhibitors (3-methyladenine (3-MA), wortmannin (Wort) and bafilomycin A1 (Baf A1)). After optimizing the inhibitor concentrations and time points (Fig. S10), we found that the autophagosome inhibitors (3-MA, Wort and Baf A1) nearly completely blocked WDR23-mediated TRAF6 degradation, whereas the ubiquitin‒proteasome inhibitors (MG132 and MG115) had no effect (Fig. 4f). Additionally, when TRAF6 protein levels were preserved using the autophagosome inhibitor Baf A1 in a ubiquitination assay, TRAF6 autoubiquitination levels remained unchanged, regardless of WDR23 expression (Fig. 4g). These findings demonstrate that WDR23 does not influence TRAF6 autoubiquitination. Taken together, these results suggest that WDR23 promotes TRAF6 degradation via an autophagosomal pathway rather than the ubiquitin–proteasome pathway.

### WDR23 deficiency enhances RANKL-induced osteoclastogenesis by increasing TRAF6 protein levels

**Fig. 5:**
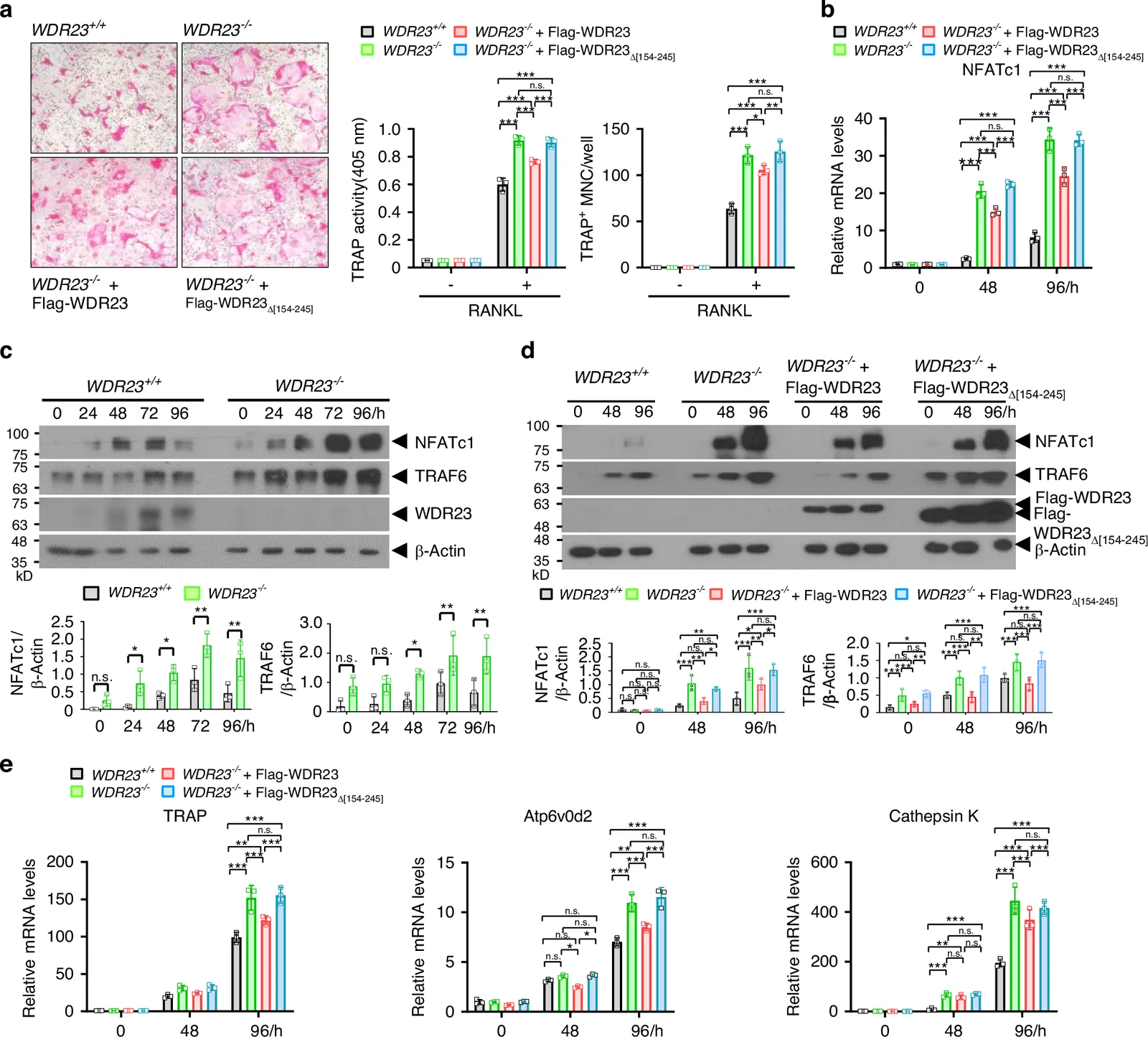
Forced expression of WDR23 in *WDR23*^*−*/*−*^ BMMs restores RANKL-induced osteoclastogenesis. **a** Restoration of osteoclastogenesis in *WDR23*^*−*/*−*^-BMMs by Flag-WDR23 but not by Flag-WDR23_Δ[154-245]_. Puromycin-resistant *WDR23*^*−*/*−*^-BMMs expressing Flag-WDR23 or Flag-WDR23_Δ[154-245]_ were differentiated into BMOCs. Osteoclastogenesis was assessed by TRAP staining (original magnification ×50) and quantified by a TRAP activity assay and by counting TRAP-positive multinucleated OCs (TRAP^+^ MNCs (≥three nuclei)). **b** Restoration of NFATc1 mRNA expression in *WDR23*^*−*/*−*^-BMMs by Flag-WDR23 during osteoclastogenesis. NFATc1 expression was measured by real-time PCR. **c** Increased NFATc1 protein expression and enhanced TRAF6 stability in *WDR23*^*−*/*−*^-BMOCs. The relative levels of NFATc1 and TRAF6 were normalized with β-actin. **d** Restoration of NFATc1 and TRAF6 protein levels in *WDR23*^*−*/*−*^-BMMs by Flag-WDR23. **e** Restoration of the expression of osteoclastogenic markers in *WDR23*^*−*/*−*^-BMMs by Flag-WDR23. The expression of osteoclastogenic markers was measured by real-time PCR. The data are shown as the means ± SD. ^*^P < 0.05. ^**^P < 0.01. ^***^P < 0.001. n.s., not significant

To investigate the in vivo effects of WDR23 on RANKL**-**induced osteoclastogenesis, *WDR23*^*−*/*−*^ mice were generated using the CRISPR-Cas9 system. The CRISPR-Cas9 targeting site at amino acid 18 on exon 2 of the WDR23 genomic locus was validated by DNA sequencing and genomic PCR analysis (Fig. S11A, B). In RANKL-induced osteoclastogenesis, WDR23 deficiency significantly increased OC formation (Fig. S11C, top), with a 1.24-fold increase in TRAP activity (1.23 ± 0.1 vs. 0.99 ± 0.07) and a 2.0-fold increase in the number of TRAP^+^ multinucleated OCs (125.3 ± 7.6 vs. 63.0 ± 8.5) compared with those in wild-type (*WDR23*^*+*/*+*^) controls (Fig. S11C, bottom). Additionally, *WDR23*^*−*/*−*^ BMOCs exhibited elevated expression levels of NFATc1 and its downstream OC markers, including TRAP, Atp6v0d2 and cathepsin K, relative to those of *WDR23*^*+*/*+*^ BMOCs (Fig. S11D, E). Consistent with the TRAF6 stability assay results (Fig. S5), compared with wild-type control BMOCs, *WDR23*^*−*/*−*^ BMOCs presented markedly increased NFATc1 and TRAF6 protein levels during RANKL-induced osteoclastogenesis (Fig. 5c). However, TRAF6 mRNA levels remained unchanged between *WDR23*^*+*/*+*^ and *WDR23*^*−*/*−*^ BMOCs, indicating that the effect of WDR23 deficiency on TRAF6 is posttranscriptional (Fig. S9C). These findings suggest that WDR23 deficiency enhances RANKL-induced osteoclastogenesis by stabilizing and increasing TRAF6 protein levels. To test whether WDR23 re-expression could restore TRAF6 protein levels and suppress RANKL-induced osteoclastogenesis in *WDR23*^*−*/*−*^ BMMs, Flag-WDR23 or a TRAF6-nonbinding Flag-WDR23_Δ[154-245]_ mutant was introduced using retroviral gene transfer and puromycin selection. Following differentiation into BMOCs, both TRAP activity assays and TRAP^+^ multinucleated OC counts revealed that the enhanced osteoclastogenesis in *WDR23*^*−*/*−*^ BMOCs was significantly suppressed by Flag-WDR23 re-expression but not by the TRAF6-nonbinding mutant (Fig. 5a). Consistently, the increase in the levels of NFATc1 and its downstream OC markers in *WDR23*^*−*/*−*^ BMOCs was inhibited by Flag-WDR23 re-expression, whereas the Flag-WDR23_Δ[154-245]_ mutant had no effect (Fig. 5b, d, e). Furthermore, Flag-WDR23 re-expression restored TRAF6 protein levels in *WDR23*^*−*/*−*^ BMOCs, but the TRAF6-nonbinding mutant failed to do so (Fig. 5d). Taken together, these results demonstrate that the interaction between TRAF6 and WDR23 is crucial for regulating RANKL-induced osteoclastogenesis by modulating TRAF6 protein levels.

### The interaction of TRAF6 and WDR23 promotes the autophagy-dependent degradation of TRAF6

**Fig. 6:**
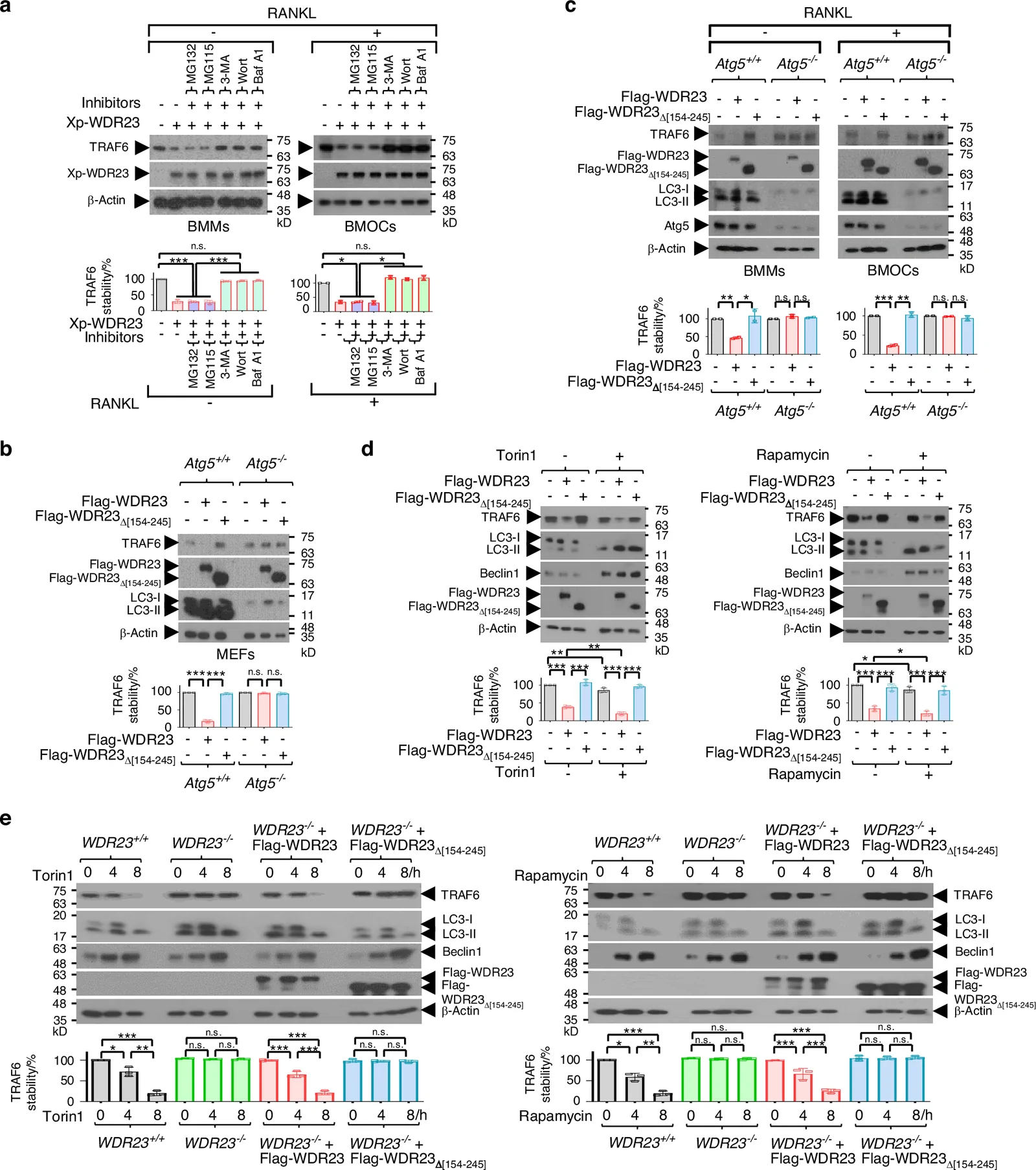
WDR23 promotes autophagy-dependent degradation of TRAF6. **a** WDR23-mediated TRAF6 degradation is rescued by autophagy inhibitors. BMMs expressing Xp-WDR23 were treated in the absence (−) or presence (+) of RANKL stimulation with ubiquitin‒proteasome inhibitors (MG132; 20 μmol/L and MG115; 20 μmol/L) or autophagy inhibitors (3-MA; 5 mmol/L, Wort; 5 μmol/L or Baf A1; 1 μmol/L) for 8 h. The relative levels of endogenous TRAF6 were normalized to β-actin. **b** WDR23-mediated TRAF6 degradation is blocked in autophagy-deficient *ATG5*^*−*/*−*^-MEFs. *ATG5*^*+*/*+*^- and *ATG5*^*−*/*−*^-MEFs expressing Flag-WDR23 or Flag-WDR23_Δ[154-245]_ were analyzed by immunoblotting, and the relative levels of TRAF6 were normalized with β-actin. **c** WDR23-mediated TRAF6 degradation is blocked in autophagy-deficient *ATG5*^*−*/*−*^-BMMs. *ATG5*^*+*/*+*^- and *ATG5*^*−*/*−*^-BMMs expressing Flag-WDR23 or Flag-WDR23_Δ[154-245]_ were analyzed by immunoblotting. **d** Autophagy induction enhances WDR23-mediated TRAF6 degradation. BMMs expressing Flag-WDR23 or Flag-WDR23_Δ[154-245]_ were treated with autophagy inducers (torin1, 100 nmol/L; rapamycin, 50 μmol/L) for 8 h. **e** Effects of the TRAF6-WDR23 interaction in autophagy inducer-stimulated BMMs. *WDR23*^*+*/*+*^-, *WDR23*^*−*/*−*^-, Flag-WDR23-expressing *WDR23*^*−*/*−*^- and Flag-WDR23_Δ[154-245]_-expressing *WDR23*^*−*/*−*^-BMMs were treated with autophagy inducers [torin1 (100 nmol/L) and rapamycin (50 μmol/L)] for the indicated times. ^*^P < 0.05. ^**^P < 0.01. ^***^P < 0.001. n.s., not significant

To investigate the mechanism underlying WDR23-mediated TRAF6 degradation, we analyzed the effects of specific inhibitors targeting either the ubiquitin–proteasomal pathway or the autophagosomal pathway. The optimal treatment conditions for increasing the inhibitor concentration and duration were established in BMMs (Fig. S12A–D). Consistent with the results observed in 293T cells (Fig. 4f), the inhibition of autophagy with 3-MA, Wort and Baf A1 effectively blocked WDR23-mediated TRAF6 degradation in both BMMs and BMOCs, while ubiquitin‒proteasome inhibitors (MG132 and MG115) had no observable effect (Fig. 6a). Consistent with these findings, basal TRAF6 protein levels were modestly elevated by autophagy inhibitors in BMMs and BMOCs lacking Xp-WDR23 overexpression (Fig. S12E). To further elucidate the role of autophagy in WDR23-mediated TRAF6 degradation, we utilized Atg5-deficient mouse embryonic fibroblasts (*Atg5*^*−*/*−*^ MEFs), which are defective in autophagosome formation and unable to convert LC3-I to LC3-II. Retroviral transduction of Flag-WDR23 into *Atg5*^*+*/*+*^ MEFs resulted in a significant reduction in endogenous TRAF6 protein levels, whereas no such reduction was observed in the *Atg5*^*−*/*−*^ MEFs (Fig. 6b). Notably, retroviral transduction of a TRAF6-nonbinding mutant (Flag-WDR23_Δ[154-245]_) failed to induce TRAF6 degradation in both the *Atg5*^*+*/*+*^ and the *Atg5*^*−*/*−*^ MEFs (Fig. 6b). These findings were further validated during osteoclastogenesis using *Atg5*^*−*/*−*^ BMMs generated via a lentiviral CRISPR–Cas9 system, which achieved approximately 92% reduction in Atg5 expression (Fig. 6c). Consistent with the *Atg5*^*−*/*−*^ MEF data in Fig. 6b, Flag-WDR23 significantly reduced endogenous TRAF6 protein levels in *Atg5*^*+*/*+*^ BMMs and BMOCs, but not in their *Atg5*^*−*/*−*^ counterparts (Fig. 6c). In contrast, the TRAF6-nonbinding WDR23 mutant did not affect TRAF6 protein levels in either genotype (Fig. 6c). Collectively, these findings suggest that WDR23 mediates TRAF6 degradation through an autophagosome-dependent pathway rather than through the ubiquitin‒proteasome pathway.

To explore the effects of autophagy activation, we treated BMMs with autophagy inducers and optimized the treatment conditions by monitoring autophagy markers, including LC3-II conversion and beclin-1 expression (Fig. S13). Under autophagy-inducing conditions (torin1 at 100 nmol/L or rapamycin at 50 μmol/L), the expression of Flag-WDR23 significantly promoted the degradation of TRAF6 compared with that in untreated controls, whereas the TRAF6 nonbinding mutant (Flag-WDR23_Δ[154-245]_) had no effect on TRAF6 stability (Fig. 6d). Consistent with these results, immunofluorescence analysis revealed that TRAF6 extensively colocalized (~72%) with LC3-positive puncta in BMMs expressing Flag-WDR23 under autophagy-inducing conditions, whereas colocalization was significantly reduced (~20%) in cells expressing Flag-WDR23_Δ[154-245]_ (Fig. S14). In *WDR23*^*−*/*−*^ BMMs, TRAF6 levels remained unchanged regardless of autophagy induction, whereas in *WDR23*^*+*/*+*^ BMMs, autophagy activation markedly enhanced TRAF6 degradation, indicating that WDR23 deficiency impairs autophagy-dependent TRAF6 degradation (Fig. 6e). In rescue experiments, re-expression of Flag-WDR23 in *WDR23*^*−*/*−*^ BMMs successfully restored autophagy-mediated TRAF6 degradation, whereas the TRAF6 nonbinding mutant failed to rescue this effect (Fig. 6e). Collectively, these findings indicate that the interaction between TRAF6 and WDR23 is essential for the autophagic recruitment and subsequent degradation of TRAF6, highlighting the critical role of WDR23 in regulating TRAF6 turnover through autophagy.

### *WDR23*^*−*/*−*^ mice exhibit an osteoporotic-like bone phenotype characterized by elevated OC formation and reduced bone formation

**Fig. 7:**
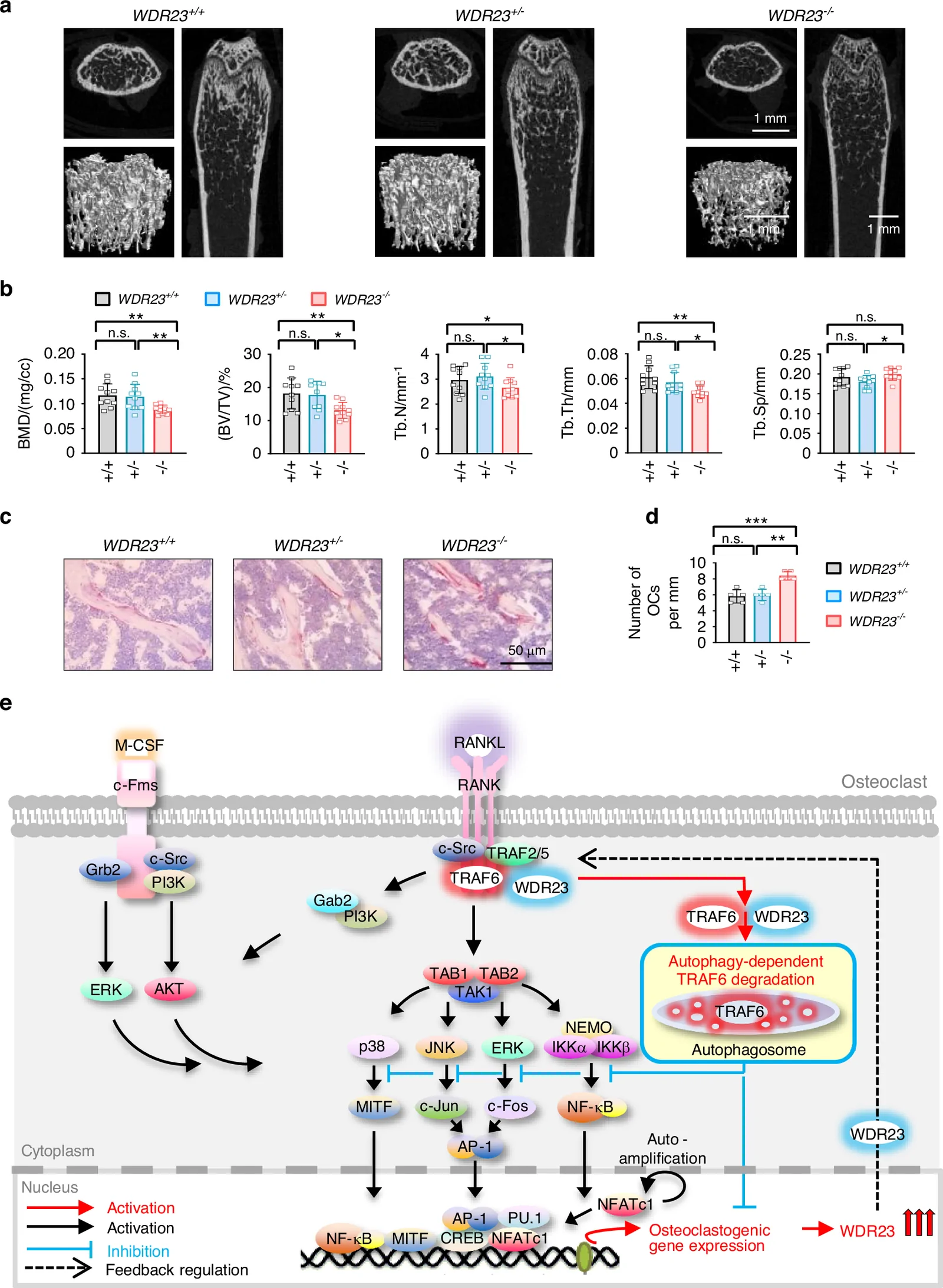
*WDR23*^*−*/*−*^ mice exhibit an osteoporotic-like bone phenotype. **a** Three-dimensional microstructural analysis of distal femurs from *WDR23*^*−*/*−*^ mice and controls. Femurs from *WDR23*^*−*/*−*^ mice (*n* = 10/group, 8-week-old females) were fixed in 10% formaldehyde and analyzed (scale bar, 1 mm) by micro-CT. *WDR23*^*+*/*+*^ and *WDR23*^*+*/^^−^ littermates were used as control mice. **b** Quantitative analysis of trabecular bone parameters in the distal femurs of *WDR23*^*−*/*−*^ mice. Trabecular structural parameters, including bone mineral density (BMD), the trabecular bone volume fraction (BV/TV), the trabecular number (Tb.N), the trabecular thickness (Tb.Th) and the trabecular spacing (Tb.Sp), were analyzed by micro-CT. **c** Histological analysis of OCs in the distal femurs of *WDR23*^*−*/*−*^ mice. OCs in paraffin-embedded femur sections were visualized by TRAP staining with hematoxylin counterstaining (scale bar, 50 μm). **d** Quantification of TRAP-positive OCs in the distal femurs of *WDR23*^*−*/*−*^ mice. **e** Proposed model of the negative feedback regulation of WDR23 in RANKL/RANK/TRAF6-mediated osteoclastogenesis. The data are shown as the means ± SD. ^*^P < 0.05. ^**^P < 0.01. ^***^P < 0.001. n.s., not significant

To investigate the impact of WDR23 deficiency on bone microarchitecture and structural integrity, we performed comparative analyses of femurs from *WDR23*^*+*/*+*^, *WDR23*^*+*/*−*^ and *WDR23*^*−*/*−*^ mice via microcomputed tomography (micro-CT) and bone histomorphometry. Three-dimensional micro-CT imaging revealed a significant reduction in bone volume and quality in the *WDR23*^*−*/*−*^ mice compared with the *WDR23*^*+*/*+*^ and *WDR23*^*+*/*−*^ controls (Fig. 7a). Quantitative analysis of bone structural parameters revealed a marked reduction in bone mineral density (BMD) in *WDR23*^*−*/*−*^ mice, with decreases ranging from 22.4% to 24.2% relative to those in *WDR23*^*+*/*+*^ and *WDR23*^*+*/*−*^ mice (Fig. 7b, first panel). Consistent with these findings, additional bone structural parameters, including the trabecular bone volume fraction (BV/TV), trabecular number (Tb.N), trabecular thickness (Tb.Th) and trabecular spacing (Tb.Sp), were significantly correlated with BMD when *WDR23*^*−*/*−*^ mice were compared with *WDR23*^*+*/*+*^ and *WDR23*^*+*/^^*−*^ controls (Fig. 7b, second through fifth panels). Notably, BV/TV, Tb.N and Tb.Th were reduced by 27.9%, 14.6% and 19.2%, respectively, whereas Tb.Sp increased by 10.4% in the femurs of the *WDR23*^*−*/*−*^ mice compared with those of the *WDR23*^*+*/^^*−*^ controls (Fig. 7b, second through fifth panels). These findings were consistent with histological evidence showing reduced trabecular bone extent in *WDR23*^*−*/*−*^ mice compared with controls (Fig. 7c). To determine the underlying mechanism of bone loss, we quantified the number of OCs in the trabecular region using TRAP staining and hematoxylin counterstaining. The results revealed a significant increase (40.0%–44.8%) in the number of TRAP^+^ OCs in the *WDR23*^*−*/*−*^ mice compared with the control mice (Fig. 7d), indicating that WDR23 deficiency promotes OC formation. We next examined whether the reduced bone mass in *WDR23*^*−*/*−*^ mice was also associated with impaired bone formation. Dynamic histomorphometric analysis via calcein double labeling revealed a marked reduction in bone formation in *WDR23*^*−*/*−*^ mice (Fig. S15A (left)). Consistently, key indices of OB function in vivo, including mineralizing surface per bone surface (MS/BS), mineral apposition rate (MAR) and bone formation rate per bone surface (BFR/BS), were all significantly decreased in *WDR23*^*−*/*−*^ mice (Fig. 15A (right)). Although WDR23 expression was detectable at low levels in primary OBs, its expression was not significantly altered by parathyroid hormone (PTH) stimulation (Fig. S15B). Nevertheless, primary OBs derived from *WDR23*^*−*/*−*^ mice exhibited diminished bone-forming capacity and downregulated expression of osteogenic markers, including osteocalcin and osteopontin, under both basal conditions and following PTH stimulation (Fig. S15C, D), indicating compromised OB function in the absence of WDR23. Taken together, these results demonstrate that WDR23 deficiency leads to an osteoporotic-like bone phenotype characterized by increased OC formation and reduced OB-mediated bone formation.

## Discussion

Our study identified WDR23 as a novel binding partner of TRAF6 that functions as a negative regulator of osteoclastogenesis by promoting the autophagic degradation of TRAF6 (Fig. 7e). These findings prompted further investigation into the precise mechanism by which WDR23 mediates TRAF6 degradation. Autophagy and the ubiquitin‒proteasome system are generally considered the most crucial protein degradation pathways involved in maintaining cellular homeostasis.^42^ Notably, most TRAF family members, including TRAF6, primarily undergo degradation via the ubiquitin–proteasome system in response to various cellular stimuli.^19^ K^48^-linked ubiquitination generally targets TRAF proteins for proteasomal degradation, whereas K^63^-linked ubiquitination is typically associated with signaling pathway activation.^11,19^ TRAF6, through its E3 ubiquitin ligase activity, plays a critical role in initiating and regulating cellular autophagy in response to diverse cellular stimuli.^26,28^ By promoting K^63^-linked ubiquitination of unc-51-like kinase 1 (ULK1, a key regulator of autophagy), TRAF6, along with autophagy and beclin-1 regulator 1 (AMBRA1), enhances ULK1 stability, self-association and functionality during the initiation of autophagy.^43^ Additionally, TRAF6 catalyzes the K^63^-linked ubiquitination of beclin-1, a downstream target of ULK1, facilitating autophagosome formation.^44^ This ubiquitination process is finely regulated by deubiquitinating enzymes such as A20 and ubiquitin-specific peptidase 15, which counteract TRAF6’s ubiquitination activities.^44,45^ Moreover, TRAF6 modulates autophagosome formation in response to oxidative stress by catalyzing the K^48^/K^63^-linked ubiquitination of Atg9A, enabling its interaction with beclin-1.^27^ TRAF6 also mediates K^63^-linked ubiquitination of LC3B, promoting the formation of the LC3B-Atg7 complex, a crucial step in autophagy progression.^46^ Furthermore, TRAF6 deficiency leads to impaired autophagy, underscoring its role as a positive regulator of autophagy induction.^47,48^ These findings suggest that TRAF6 serves as a critical molecular hub, coordinating autophagic responses across various physiological and pathological contexts.

The CUL4A-DDB1 E3 ubiquitin ligase complex also regulates autophagy induction by modulating AMBRA1 levels through K^48^-linked ubiquitination and subsequent proteasomal degradation in response to autophagy stimuli.^49,50^ Furthermore, AMBRA1 stability during autophagy induction is regulated via its interaction with DDB1 in a ULK1/TRAF6-dependent manner.^49^ WDR23 functions as a substrate receptor within the CUL4A-DDB1 E3 ubiquitin ligase complex, facilitating ubiquitin-dependent proteasomal degradation of cellular mediators involved in the stress response, the cell cycle, cell division and longevity.^29,30,34,35,38,50^ Among its known downstream targets, the CUL4-DDB1-WDR23 E3 ubiquitin ligase complex promotes the ubiquitin–proteasome-mediated degradation of NRF2.^30,34,51^ NRF2 acts as a negative regulator of osteoclastogenesis, particularly under oxidative stress conditions; however, the regulation of WDR23 expression under physiological contexts, including oxidative stress and RANKL signaling, remains poorly defined.^52,53^ Structurally, WDR23 interacts with the CUL4A-DDB1 E3 ubiquitin ligase complex through two DxR consensus binding motifs located within WD40 repeat domains 4 and 5.^29^ However, our data revealed that TRAF6 stability was unaffected by the expression of WDR23_R338H, R385H_, which is a DxR substitution mutant (Fig. S16), indicating that the canonical E3 ubiquitin ligase activity of the CUL4A-DDB1-WDR23 complex is not involved in the degradation of TRAF6. Additionally, inhibiting the ubiquitin‒proteasome system with agents such as MG132 and MG115 did not impact the autophagy-dependent degradation of TRAF6 (Figs. 4f and 6a). These findings highlight the existence of a unique regulatory mechanism by which WDR23 interacts with TRAF6 to regulate its stability, which is distinct from the proteasomal degradation pathway.

TRAF6 autoubiquitination represents an important regulatory mechanism in signal transduction, whereby K^63^-linked chains facilitate downstream signaling, whereas K^48^-linked chains mediate ubiquitin–proteasomal degradation of TRAF6.^11,18,19^ However, a previous study demonstrated that a lysine-deficient TRAF6 mutant (TRAF6_∆K_) is capable of rescuing RANKL-mediated signaling and osteoclastogenesis in *TRAF6*^*−*/*−*^-BMMs, indicating that TRAF6 autoubiquitination per se is not a prerequisite for OC differentiation.^54^ Consistent with this observation, our data show that the stability of the TRAF6_∆K_ mutant was significantly diminished through its interaction with WDR23 (Fig. 4d, e). Collectively, these findings indicate that TRAF6 autoubiquitination is dispensable for TRAF6 degradation during RANKL-mediated osteoclastogenesis. Interestingly, recent studies have identified key regulatory pathways that modulate TRAF6 activity during autophagy, thereby enhancing our understanding of its role as a central regulator in response to diverse cellular stimuli. These regulatory pathways include protein-protein interactions that either enhance or inhibit the role of TRAF6 in autophagy. For example, TRAF6 interacts with the key lysosomal protein Rab7 via its RING finger domain, triggering K^63^-linked ubiquitination of Rab7 to promote autophagosome‒lysosome fusion during *Mycobacterium* infection.^47^ Conversely, TRAF6 E3 ligase activity can be inhibited by interaction with peroxiredoxin 1, which targets the RING finger domain of TRAF6, reducing beclin-1 ubiquitination and suppressing autophagy.^55^ TRAF6 also undergoes autophagy-mediated degradation in cervical cancer cells through its interaction with DAP kinase-related apoptosis-inducing kinase 1 via its TRAF domain.^56^ Additionally, the interaction of avibirnavirus VP3 with TRAF6, which targets the zinc finger domain, facilitates the autophagic degradation of TRAF6 during viral infection.^22^ Another important regulatory mechanism involves post-translational modifications of TRAF6, such as phosphorylation and ubiquitination, which can fine-tune its activity. For example, glycogen synthase kinase-3β phosphorylates TRAF6 at T266, promoting its K^48^-linked ubiquitination and subsequent proteasomal degradation, thereby attenuating TRAF6-mediated autophagic degradation of CTNNB1/β-catenin during cancer progression.^46^ Conversely, IKKε-mediated phosphorylation of TRAF6 at S129, S188, S268, S279 and S324 prevents its autophagic degradation in response to LPS stimulation.^21^ Within these regulatory pathways of TRAF6 in autophagy, our study demonstrated that WDR23 acts as a key regulator of TRAF6 stability through protein‒protein interactions. The TRAF domain of TRAF6 is necessary for its interaction with the WD40 repeat domains 1 and 2 of WDR23, thereby promoting the autophagy-dependent degradation of TRAF6 during RANKL-induced osteoclastogenesis (Fig. 2). Previous studies have shown that the TRAF domain of TRAF6 typically interacts with the consensus TRAF6-binding motif (PxExxZ (Z: an acidic or aromatic residue)) in its binding partners.^16^ WDR23 has a putative PxExxZ motif within its WD40 repeat domain 3 (residues 261–266). However, our data indicate that this putative PxExxZ motif in WDR23 is not essential for the interaction (Figs. 1e, g and S17). Interestingly, we found that among the TRAF family members, only TRAF6 undergoes WDR23-mediated autophagy-dependent degradation, despite the ability of WDR23 to interact with other TRAF family members (Figs. S1C and S7). These findings suggest that WDR23 selectively promotes the autophagy-dependent degradation of TRAF6 within the TRAF family. Therefore, on the basis of our findings and those of previous studies, it is plausible that an additional cellular factor selectively targeting the TRAF6-WDR23 interaction may contribute to the WDR23-mediated degradation of TRAF6. To elucidate the molecular mechanisms underlying WDR23-mediated TRAF6 degradation, further investigations are needed to identify additional cellular factors that may modulate WDR23 function.

Beyond its established role in osteoclastogenesis, TRAF6 also participates in multiple signaling pathways, including bone morphogenetic protein (BMP), transforming growth factor-β and inflammatory signaling cascades, that regulate OB differentiation and function.^1,57,58^ Notably, the significant reduction in bone formation observed in *WDR23*^*−*/*−*^ mice strongly suggests that WDR23 may contribute to the regulation of OB activity in vivo (Fig. S15). Given that WDR23-mediated autophagy-dependent degradation selectively targets TRAF6, it is plausible that this regulatory axis may also influence OB lineage commitment and bone-forming capacity. Although our current study primarily focuses on RANKL-induced osteoclastogenesis, the potential impact of the WDR23–TRAF6 pathway on OB proliferation, differentiation and matrix mineralization remains to be determined. Future investigations should determine whether WDR23-mediated regulation of TRAF6 alters downstream osteogenic signaling networks, including transcriptional and translational programs that govern OB differentiation and function. Such investigations will be important to clarify the contribution of the WDR23–TRAF6 axis to the coupling process between bone resorption and bone formation.

In summary, our study demonstrated that WDR23 is a novel TRAF6 binding partner that acts as a crucial negative feedback regulator of RANKL-induced osteoclastogenesis. The interaction of WDR23 with TRAF6 reduced TRAF6 stability through an autophagy-dependent pathway. *WDR23*^*−*/*−*^ mice exhibited an osteoporotic phenotype characterized by increased OC formation and reduced bone mass. These findings provide novel insights into TRAF6 regulation, emphasizing its importance in bone homeostasis and its potential as a therapeutic target for bone-related diseases associated with aberrant OC formation and function.

## Materials and methods

### Cell lines and mice

Plat-E (retrovirus packaging cell line) and 293T (human embryonic kidney cell line) cells were cultured in DMEM (Welgene, Daegu, Korea) supplemented with 10% FBS (Thermo Fisher Scientific) and an antibiotic/antimycotic solution (Welgene) at 37 °C in a 5% CO_2_ atmosphere. BMMs and RAW 264.7 cells (murine monocytic cell line) were cultured in α-MEM (Thermo Fisher Scientific) supplemented with 10% FBS. Atg5 wild-type (*Atg5*^*+*/*+*^) and knockout (*Atg5*^*−*/*−*^) mouse embryonic fibroblasts (MEFs) were maintained in DMEM supplemented with 15% FBS.^59^ *Atg5*^*−*/*−*^ BMMs were generated using a lentiviral CRISPR–Cas9 system as described previously.^60^ *WDR23*^*−*/*−*^ mice were generated via the CRISPR‒Cas9 system with a specific guide RNA sequence (5′-TCTTCGGGACAAGCCCTCAGAGG-3′) from Macrogen (Seoul, Korea). C57BL/6J mice were purchased from Daehan Biolink Co. (Umsung, Korea) and maintained under pathogen-free conditions. All animal studies were approved (202103A-CNU-083, 202209A-CNU-145, 202203A-CNU-006 and 202401A-CNU-008) by the Animal Experiment Ethics Committee of Chungnam National University.

### Plasmids

Epitope-tagged eukaryotic expression plasmids for WDR23, TRAF6 and its mutants were generated by PCR-based subcloning into the following vectors: pcDNA3.1-His (Thermo Fisher Scientific), pEF4/Myc-His (Thermo Fisher Scientific), pFlag-CMV2 (Sigma‒Aldrich), pEBG^17^ and pMX-puro-Flag.^61^ The expression plasmids for Flag-TRAF6, Flag-TRAF6_C70A_ (E3 ubiquitin ligase activity-defective mutant), Flag-TRAF6_∆K_ (lysine-deficient TRAF6: all K residues in TRAF6 mutated to R) and HA-Ub were described previously.^17^ The plasmids p(κB)_3_-NF-κB-luciferase (NF-κB-Luc), pAP-1-luciferase (AP-1-Luc) and pcDNA3.0HisLacZ (Thermo Fisher Scientific) were described previously.^17^ The pmRFP-LC3 plasmid (Addgene #21075, Watertown, MA, USA) was modified by PCR-based subcloning into the pMX-puro-Flag vector. The specific short hairpin RNA (shRNA) sequences targeting *WDR23*^*KD*^ were as follows: (sense) 5′-GATCCCGGAAGAGGTGCAAATTTTCAAGAGAAATTTGCTGCACCTCTTCCTTTTTA-3′ and (antisense) 5′-AGCTTAAAAAGGAAGAGGTGCAGCAAATTTCTCTTGAAAATTTGCACCTCTTCCGGG-3′). These sequences were subsequently subcloned and inserted into pSUPER.retro.puro (OligoEngine, Seattle, WA). GFP^KD^ was used as an empty vector control as described previously.^62^

### Protein–protein interaction

TRAF6-interacting proteins were analyzed by proteomic approaches as described previously.^62^ Briefly, 293T cells plated at 5 × 10^6^ cells per 100-mm plate were transfected with Flag-TRAF6 using TurboFect transfection reagent (Thermo Fisher Scientific). At 24 h posttransfection, the transfected cells were lysed in lysis buffer (20 mmol/L Tris-HCl (pH 7.5), 150 mmol/L NaCl, 10% glycerol, 1 mmol/L EDTA, 1% Triton X-100, 2 mmol/L sodium orthovanadate, 2 mmol/L NaF and 1× proteinase inhibitor cocktail). After centrifugation, the Flag-TRAF6-bound proteins were immunoprecipitated with anti-Flag beads for 16 h at 4 °C, after which the bound proteins were washed three times with lysis buffer. Flag-TRAF6-interacting proteins were analyzed by nanoscale liquid chromatography coupled with tandem mass spectrometry. The protein interaction between TRAF6 and WDR23 was analyzed as previously described.^62^ Briefly, 293T cells (8 × 10^5^ cells/well) plated in 6-well plates were cotransfected with epitope- or GST-tagged TRAF6 and WDR23 using TurboFect transfection reagent. At 24 h posttransfection, the cells were lysed in cell lysis buffer. The target protein was pulled down or immunoprecipitated using GST beads or anti-Flag beads. Protein‒protein interactions were analyzed by immunoblotting with antibodies against GST, Xp, Myc and Flag. For endogenous protein‒protein interactions, endogenous TRAF6 or WDR23 proteins in 293T cells or BMMs were immunoprecipitated using protein G beads with an anti-TRAF6 or anti-WDR23 antibody for 12 h, followed by immunoblotting with antibodies against TRAF6 or WDR23.

### Osteoclastogenesis

Osteoclastogenesis was performed as previously described.^17,63,64^ Briefly, bone marrow cells isolated from the femurs of 8-week-old male C57BL/6J mice were differentiated into BMMs with 150 ng/mL M-CSF treatment in α-MEM supplemented with 10% FBS for 2 d. To prepare WDR23-overexpressing or *WDR23*^*KD*^ BMMs, retroviral supernatants were prepared from Plat-E cells (2 × 10^6^ cells/dish) transfected with retroviral expression plasmids using TurboFect transfection reagent (Thermo Fisher Scientific) as previously described.^17^ BMMs (3 × 10^5^ cells/dish) were infected with retroviral supernatant harboring WDR23-overexpressing or *WDR23*^*KD*^ cassettes with polybrene (8 μg/mL) for 6 h, and then, the infected BMMs were selected with puromycin (2 μg/mL) in the presence of M-CSF (150 ng/mL) for 2 d. Puromycin-resistant BMMs (2 × 10^4^ cells/well) were differentiated into BMOCs in 96-well plates in the presence of M-CSF (50 ng/mL) and RANKL (200 ng/mL) for 4 d. RAW 264.7 cells (2 × 10^3^ cells/well) were differentiated into OCs in 96-well plates in the presence of RANKL (200 ng/mL) for 4 d as described previously.^17^ OCs were further analyzed by TRAP assays, immunoblotting analysis and real-time PCR analysis, as described previously.^17,63,64^

### Luciferase reporter assay and ubiquitination assay

Luciferase assays were performed as described previously.^17^ Briefly, 293T cells (1.25 × 10^5^ cells/well) in 24-well plates were cotransfected with a luciferase reporter (0.25 μg), various expression plasmids (0.15–2.0 μg) and pcDNA3.0HisLacZ (0.15 μg). At 24 h posttransfection, the luciferase activities were measured using a luciferase assay kit (Promega, Madison, WI, USA) and normalized to the β-galactosidase activity using a β-galactosidase assay kit (Thermo Fisher Scientific). The TRAF6 ubiquitination assay was performed as previously described.^17^ Briefly, 293T cells (8 × 10^5^ cells/well) were seeded in 6-well plates and cotransfected with Flag-TRAF6 (0.5 μg), HA-Ub (0.5 μg) or Xp-WDR23 (0.5 μg). To inhibit autophagy, the cells were treated with the autophagy inhibitor Baf A1 (1 μmol/L) for 8 h prior to harvesting. At 24 h posttransfection, the cells were subjected to immunoprecipitation and immunoblotting analysis with specific antibodies to assess TRAF6 ubiquitination.

### Immunofluorescence analysis

Immunofluorescence analysis was performed as described previously.^17,62^ Briefly, BMMs (1.0 × 10^5^ cells/mL) co-expressing Flag-WDR23 or Flag-WDR23_Δ[154-245]_ along with LC3-RFP were plated onto cell culture slides and treated with either torin1 (100 nmol/L) or rapamycin (50 μmol/L) for 8 h. Immunostaining was carried out overnight at 4 °C using a rabbit anti-TRAF6 antibody (Cell Signaling Technology), followed by an Alexa Fluor 633-conjugated anti-rabbit IgG secondary antibody (Thermo Fisher Scientific). Flag-WDR23 expression was detected using a mouse anti-Flag antibody (Sigma-Aldrich), followed by a FITC-conjugated anti-mouse IgG secondary antibody (Jackson ImmunoResearch, West Grove, PA). The nuclei were counterstained with DAPI. Co-localization of TRAF6 and LC3-RFP within autophagosomal puncta was visualized using a Zeiss LSM 800 confocal microscope equipped with ZEN imaging software (Zeiss, Jena, Germany).

### Bone phenotype analysis

Bone phenotype analysis was performed as previously described.^63^ Briefly, femurs from *WDR23*^*+*/*+*^, *WDR23*^*+*/^^*−*^ and *WDR23*^*−*/*−*^ mice (8-week-old females, *n* = 10 per group) were fixed with 10% formaldehyde. For microcomputed tomography (micro-CT) analysis, trabecular morphometry of distal femurs was performed using SkyScan 1076 (Bruker micro-CT, Kontich, Belgium). The bone parameters were analyzed by measuring the bone mineral density (BMD), trabecular bone volume fraction (BV/TV), trabecular number (Tb.N), trabecular thickness (Tb.Th) and trabecular spacing (Tb.Sp) in the region of interest using 50 slices approximately 0.4 mm away from the growth plate of the distal femur. For histological analysis, the fixed femurs were decalcified in 15% EDTA solution at room temperature for 31 d prior to being embedded in paraffin. Paraffin sections were sliced (7 μm) and subjected to TRAP staining and hematoxylin counterstaining. The number of TRAP^+^ OCs in the femur was counted by visualization under a microscope. In vivo dynamic bone formation analysis was performed as previously described.^6^ Briefly, 8-week-old wild-type (*n* = 5) and *WDR23*^*−*/*−*^ mice (*n* = 5) were injected intraperitoneally with calcein (20 mg/kg body weight). A second injection was administrated 5 days later, and the mice were sacrificed 2 days after the second injection. Femurs were harvested and fixed in 4% paraformaldehyde in PBS at 4 °C for 24 h. Following fixation, femurs were decalcified, embedded in cryomolds and cryosectioned at a thickness of 5 µm. Finally, MS/BS, MAR and BFR/BS were quantified using OsteoMeasure software (Version 2.02; OsteoMetrics, TN, USA) at the Chronic Metabolic Diseases Research Center, Sookmyung Women’s University. In vitro bone formation analysis was performed as previously described.^65^ Briefly, primary OBs isolated from the calvaria of newborn wild-type and *WDR23*^***−*/*−***^ mice were cultured in α-MEM including 10% FBS and supplemented with 50 μmol/L ascorbic acid, 10 mmol/L β-glycerophosphate, 100 ng/mL BMP-2 and 10^−7^ mol/L dexamethasone for 5 d. The primary OBs were further activated by 10^−7^ mol/L PTH stimulation for 3 d. Alkaline phosphatase (ALP) activity was assessed in culture of primary OBs following stimulation with 50 μmol/L ascorbic acid, 10 mmol/L β-glycerophosphate and 10^−7^ mol/L dexamethasone for 6 d. Mineralization of cultured primary OBs was evaluated after stimulation with 50 μmol/L ascorbic acid and 10 mmol/L β-glycerophosphate for 21 d using alizarin red S (ARS) staining. Expression of OB marker genes was analyzed by real-time PCR using the following primers: osteocalcin (sense), 5'-TTACTTTATGCTTCTCAGAGC-3'; osteocalcin (antisense), 5'-AAATAGTGATACCGTAGATGC-3'; osteopontin (sense), 5'-TGCACCCAGATCCTATAGCCA-3'; osteopontin (antisense), 5’-TGTGCTCATGGCTTTCATTGG-3’.

### Statistical analysis

The data represent the means ± SDs (*n* = 3 per group). Statistical analyses were performed using a one-tailed Student’s *t* test for pairwise comparisons or two-way ANOVA for multiple comparisons. *P* values < 0.05 were considered statistically significant.

## Supplementary information

Supplementary information
