# Supplementary material for: WDR23 prevents bone loss by promoting autophagic degradation of TRAF6 in osteoclastogenesis
Source: Bone Res. 2026 Sep 10;14:98. doi: 10.1038/s41413-026-00560-2 (PMC13558766; doi:10.1038/s41413-026-00560-2)
Supplement: Supplementary file 1 [file 41413_2026_560_MOESM1_ESM.docx]

**WDR23 Prevents Bone Loss by Promoting Autophagic Degradation of TRAF6 in Osteoclastogenesis**

Hye-Won Park^1^, Jungeun Yu^1^, Jiyeon Yu^1^, Jinseon You^1^, Yeon Hee Kook^1,6^, Joo-Yong Lee^2^, Taesoo Kim^3^, Chul-Ho Lee^4^, Hee-Chung Chung^5^, Jong-Soon Choi^5^, Sangkyu Lee^6^ and Jaerang Rho^1,§^

**Supplementary Information**

**Supplementary Materials and Methods**

**Antibodies, reagents, cells and mice**

Specific antibodies were purchased from the following commercial sources: anti-Flag epitope, anti-Myc epitope and anti-β-actin from Sigma‒Aldrich (St. Louis, MO, USA); anti-Xpress (Xp) epitope from Thermo Fisher Scientific (Carlsbad, CA, USA); anti-GST and anti-NFATc1 from Santa Cruz Biotechnology (Santa Cruz, CA, USA); anti-TRAF6, anti-LC3, anti-IκBα, anti-phospho-IκBα, anti-p65, anti-phospho-p65, anti-ERK, anti-phospho-ERK, anti-p38, anti-phospho-p38, anti-JNK and anti-phospho-JNK from Cell Signaling Technology (Danvers, MA, USA); and anti-WDR23 from St John's Laboratory (London, United Kingdom). Cycloheximide (CHX), 3-methyladenine (3-MA), wortmannin (Wort), bafilomycin A1 (Baf A1), torin1, rapamycin, glutathione-Sepharose 4B (GST beads) and anti-Flag affinity gels (anti-Flag beads) were purchased from Sigma‒Aldrich. MG132 and MG115 were purchased from Calbiochem (La Jolla, CA). Protein G beads were purchased from Amersham-Pharmacia Biotech (Piscataway, NJ, USA). Recombinant human soluble RANKL and human M-CSF were described previously ^1, 2^. The generation of TRAF6^-/-^ mice was described previously ^3^.

**Plasmids**

The expression plasmids for Flag-RANK, GST-RANK_cyto_ (GST-fused RANK cytoplasmic tail), Flag-RANK-AAA (Flag-RANK lacking three TRAF6-binding consensus sites (T6BSs)), GST-RANK_cyto_-AAA (GST-RANK_cyto_ lacking T6BSs), Flag-TRAF1, Flag-TRAF2, Flag-TRAF3, Flag-TRAF5 and Flag-TRAF6 were described previously ^4^. The expression plasmids for Flag-TRAF6 (pCMV) and Flag-TRAF6 (pEF1α) were generated by PCR-based subcloning into the pFlag-CMV2 vector (Sigma‒Aldrich) and pEF4/Myc-His (Thermo Fisher Scientific), respectively. The expression plasmids used for Flag-WDR23_R338H_, Flag-WDR23_R385H_ and Flag-WDR23_R338H,R385H_ were described previously ^5^. The expression plasmid for Flag-WDR23_P261A,E263A_ was generated by PCR-based site-directed mutagenesis as described previously ^4^.

**TRAF6 stability and degradation**

TRAF6 stability was analyzed by immunoblotting with antibodies against Flag and Xp. TRAF6 degradation assays were performed as described previously ^6^. 293T cells cotransfected with Flag-TRAF6 and Xp-WDR23 were treated with ubiquitin‒proteasome inhibitors (MG132; 20 μM and MG115; 20 μM) or autophagy inhibitors (3-MA; 5 mM, Wort; 5 μM and Baf A1; 1 μM) for 8 h. BMMs (3×10^5^ cells/dish) were treated with ubiquitin‒proteasome inhibitors or autophagy inhibitors in the presence of M-CSF (50 ng/ml) for 8 h. To induce autophagy, puromycin-resistant BMMs expressing either Flag-WDR23 or Flag-WDR23_Δ[154-245]_ were treated with torin1 (100 nM) or rapamycin (50 μM) for 8 h. Cycloheximide (CHX) chase analysis was performed as described previously ^7^. Briefly, 293T cells (8×10^5^ cells/well) plated in 6-well plates were cotransfected with Flag-TRAF6 and Xp-WDR23 using TurboFect transfection reagent (Thermo Fisher Scientific). At 24 h posttransfection, the cells were lysed in cell lysis buffer. The cell lysates were incubated with or without CHX (50 μg/ml) for the indicated times.

**Immunofluorescence analysis**

Immunofluorescence analysis was performed as described previously ^4, 8^. Briefly, MEFs (0.5×10^5^ cells/ml) or BMMs (1.0×10^5^ cells/ml) expressing Flag-WDR23 or Flag-WDR23_Δ[154-245]_ were plated onto cell culture slides. After 24 and 48 h post-incubation, cells were then fixed with 4% paraformaldehyde and permeabilized with 0.1% Triton X‐100. Immunostaining was carried out overnight at 4 °C using a rabbit anti-TRAF6 antibody (Cell Signaling Technology), followed by an Alexa Fluor 633-conjugated anti-rabbit IgG secondary antibody (Thermo Fisher Scientific). Flag-WDR23 expression was detected using a mouse anti-Flag antibody (Sigma-Aldrich), followed by a FITC-conjugated anti-mouse IgG secondary antibody (Jackson ImmunoResearch, West Grove, PA). The nuclei were visualized by DAPI staining. The subcellular localization of TRAF6 and WDR23 was examined using a Zeiss LSM 800 confocal microscope with ZEN imaging software (Zeiss, Jena, Germany).

**Primers of real-time PCR analysis**

Real-time PCR were performed as described previously ^1, 2, 4^. Briefly, total RNA was isolated using TRIzol reagent (Thermo Fisher Scientific), and cDNA templates were synthesized from 1 μg of total RNA using M-MLV reverse transcriptase (Enzynomics, Daejeon, Korea). The cDNA was then subjected to real-time PCR analysis (Bio-Rad) with the specific primers for TRAF6, NFATc1, TRAP, DC-STAMP, Atp6v0d2 and cathepsin K. The specific primers used were as follows: TRAF6 (sense), 5’-TCC TGC AGC GCT GCA GTG AAA-3’; TRAF6 (antisense), 5’-TGC AAA CAG CCT TTA TTT GGG-3’; NFATc1 (sense), 5’-CCC AGT ATA CCA GCT CTG CCA TTG-3’; NFATc1 (antisense), 5’-GGA GCC TTC TCC ACG AAA ATG ACT-3’; TRAP (sense), 5’-AAA TCA CTC TTC AAG ACC AG-3’; TRAP (antisense), 5’-TTA TTG AAC AGC AGT GAC AG-3’; DC-STAMP (sense), 5’-TCC TCC ATG AAC AAA CAG TTC CAA-3’; DC-STAMP (antisense), 5’-AGA CGT GGT TTA GGA ATG CAG CTC-3’; Atp6v0d2 (sense), 5’-TTC AGT TGC TAT CCA GGA CTC GGA-3’; Atp6v0d2 (antisense), 5’-GCA TGT CAT GTA GGT GAG AAA TGT GCT CA-3’; Cathepsin K (sense), 5’-ACG GAG GCA TTG ACT CTG AAG ATG-3’; Cathepsin K (antisense), 5’-GTT GTT CTT ATT CCG AGC CAA GAG-3’; β-Actin (sense), 5’-ATG AAG ATC CTG ACC GAG CG-3’; and β-Actin (antisense), 3’-TAC TTG CGC TGA GGA GC-5’. β-Actin was used as the internal control.

**Supplementary References**

1.	Jeon H*, et al.* 1,3-Dibenzyl-5-Fluorouracil Prevents Ovariectomy-Induced Bone Loss by Suppressing Osteoclast Differentiation. *Immune Netw* **22**, e43 (2022).

2.	Yu J*, et al.* Pax5 Negatively Regulates Osteoclastogenesis through Downregulation of Blimp1. *Int J Mol Sci* **22**, (2021).

3.	Walsh MC, Kim GK, Maurizio PL, Molnar EE, Choi Y. TRAF6 autoubiquitination-independent activation of the NFkappaB and MAPK pathways in response to IL-1 and RANKL. *PloS one* **3**, e4064 (2008).

4.	Yu J*, et al.* Interaction of Tumor Necrosis Factor Receptor-associated Factor 6 (TRAF6) and Vav3 in the Receptor Activator of Nuclear Factor kappaB (RANK) Signaling Complex Enhances Osteoclastogenesis. *J Biol Chem* **291**, 20643-20660 (2016).

5.	Angers S, Li T, Yi X, MacCoss MJ, Moon RT, Zheng N. Molecular architecture and assembly of the DDB1-CUL4A ubiquitin ligase machinery. *Nature* **443**, 590-593 (2006).

6.	Busch J*, et al.* TRAF6 Phosphorylation Prevents Its Autophagic Degradation and Re-Shapes LPS-Triggered Signaling Networks. *Cancers (Basel)* **13**, (2021).

7.	Lee K, Chung YH, Ahn H, Kim H, Rho J, Jeong D. Selective Regulation of MAPK Signaling Mediates RANKL-dependent Osteoclast Differentiation. *Int J Biol Sci* **12**, 235-245 (2016).

8.	Park ES*, et al.* TDAG51 promotes transcription factor FoxO1 activity during LPS-induced inflammatory responses. *The EMBO journal* **42**, e111867 (2023).

**Supplementary Figure legends**

**Figure S1. TRAF6 interacts with WDR23. A.** List of proteins identified as TRAF6-interacting candidates. TRAF6-interacting proteins were identified through proteomic analysis following immunoprecipitation of the Flag-TRAF6 protein from 293T cell lysates. **B.** Identification of WDR23 by immunoblotting analysis. The presence of WDR23 in the immunoprecipitated (IP) samples was confirmed via anti-WDR23 immunoblotting (IB). The specific bands are indicated by arrows. **C.** WDR23 interacts with other TRAF family members. 293T cells were cotransfected with Flag-TRAFs (1.0 μg) and GST-WDR23 (1.0 μg). GST-WDR23 was pulled down (PD) using GST beads. The bound proteins were visualized via anti-Flag (top panel) immunoblotting (IB). The levels of GST-WDR23 and Flag-TRAFs were analyzed via anti-GST (middle panel) and anti-Flag IB (bottom panel), respectively. GST alone (mock) was used as a control. **D**. TRAF6-WDR23 complex formation during RANKL-mediated osteoclastogenesis. BMMs were differentiated into BMOCs via RANKL stimulation (200 ng/ml) in the presence of M-CSF (50 ng/ml) for 4 d. Endogenous TRAF6 was immunoprecipitated with anti-(α-)TRAF6 antibodies, and proteins bound to TRAF6 were visualized by IB with anti-WDR23 antibodies (WDR23_endo_, top panel). The levels of immunoprecipitated (IP) endogenous TRAF6 were visualized by IB with anti-TRAF6 antibodies (TRAF6_endo_, second panel). The levels of endogenous TRAF6 and WDR23 in whole-cell lysates were detected by anti-TRAF6 (third panel) and anti-WDR23 IB (bottom panel), respectively.

**Figure S2.** **TRAF6 interacts with WDR23 during RANKL-mediated signaling.** **A**. TRAF6 interacts with WDR23 in the RANK signaling complex. The RANK-AAA mutant lacking three TRAF6-binding consensus sites (T6BSs: substitution of glutamic acid (E) in the T6BSs with alanine (A)) is indicated above the diagram. 293T cells were transfected with Flag-tagged RANK receptor (Flag-RANK or Flag-RANK-AAA mutant (1.0 μg)), Xp-WDR23 (1.0 μg) or GST-TRAF6 (1.0 μg). Flag-RANK was immunoprecipitated using anti-Flag antibodies, and the bound proteins were visualized by anti-GST (top panel) or anti-Xp IB (second panel). The immunoprecipitated Flag-RANK protein was detected by IB with anti-Flag antibodies (third panel), and the level of GST-TRAF6 or Xp-WDR23 expression was detected by anti-Xp (fourth panel) or anti-GST IB (bottom panel), respectively. **B**. TRAF6-WDR23 interaction in the RANK cytoplasmic tail (RANK_cyto_). The RANK_cyto_-AAA mutant lacking three TRAF6-binding consensus sites (T6BSs: substitution of glutamic acid (E) in the T6BSs with alanine (A)) is indicated above the diagram. 293T cells were transfected with a GST-tagged RANK cytoplasmic tail (GST-RANK_cyto_ or GST-RANK_cyto_-AAA mutant (1.0 μg)), Xp-WDR23 (1.0 μg) or GST-TRAF6 (1.0 μg). Proteins bound to GST-RANK_cyto_ were visualized by anti-Flag (top panel) or anti-Xp IB (second panel). The GST-RANK pulled down by the GST beads was detected by anti-GST IB (third panel). The levels of Flag-TRAF6 and Xp-WDR23 expression were detected by anti-Xp (fourth panel) and anti-Flag IB (bottom panel), respectively. **C**. Restoration of osteoclastogenesis in TRAF6^-/-^-BMMs by Flag-TRAF6 deletion mutants. Macrophages derived from TRAF6^-/-^-fetal liver were transduced with retroviruses expressing Flag-TRAF6, Flag-TRAF6_[1-355]_ or Flag-TRAF6_[356-530]_ and selected with puromycin (2 μg/ml) in the presence of M-CSF (150 ng/ml) for 2 d. Puromycin-resistant TRAF6^-/-^-macrophages were further differentiated into OCs with M-CSF (50 ng/ml) and RANKL (200 ng/ml) for 4 d. The expression of Flag-TRAF6 was analyzed by immunoblotting with anti-Flag and anti-TRAF6 antibodies (left panels). Osteoclastogenesis was assessed by TRAP staining (right top panels; original magnification ×50) and quantified by a TRAP activity assay and by counting TRAP-positive multinucleated OCs (right bottom panels). The data are shown as the means ± SD. ^***^*P*<0.001. n.s., not significant.

**Figure S3.** **The TRAF6-WDR23 interaction is not blocked by the removal of a single** **WD40 repeat domain in WDR23.** **A**. Schematic diagram of the deletion of a single WD40 repeat domain in WDR23. Seven WD40 repeat domains are numbered 1-7 in the gray box. The relative binding affinities of WDR23 deletion mutants with TRAF6 are summarized on the right. **B**. The TRAF6-WDR23 interaction is not affected by deletion of the single WD40 repeat domain of WDR23. 293T cells were transfected with GST-TRAF6 (1.0 μg) with Xp-WDR23 or its deletion mutants (1.0 μg). WDR23 deletion mutants bound to GST-TRAF6 were visualized via anti-Xp immunoblotting (IB) (top panel). The levels of GST-TRAF6 determined by GST bead pulldown (PD) and Xp-WDR23 were visualized by anti-GST IB (middle panel) and anti-Xp IB (bottom panel), respectively. **C**. Schematic diagram of WDR23 and its internal deletion mutants. The interaction of WDR23 internal deletion mutants with TRAF6 are summarized on the right. **D**. WD40 repeat domains 1 and 2 of WDR23 are involved in the TRAF6-WDR23 interaction.

**Figure S4. WDR23 negatively regulates the RANK-mediated signaling pathway. A**. Downregulation of the RANKL-induced signaling pathway by forced WDR23 expression in BMMs. In the left panels, BMMs transduced with retroviruses expressing Flag-WDR23 were selected with puromycin (2 μg/ml) in the presence of M-CSF (150 ng/ml) for 2 d. Puromycin-resistant BMMs were stimulated with 200 ng/ml RANKL for the indicated times. The RANKL-induced signaling pathway was subsequently analyzed by immunoblotting with antibodies that recognize phosphorylated and total IκBα, NF-κB p65, p38, JNK and ERK. The expression levels of TRAF6 and WDR23 were analyzed by immunoblotting with anti-Flag, anti-WDR23 and anti-TRAF6 antibodies. β-Actin was used as the loading control. Mock, empty vector control. In the right panels, the relative levels of the phosphorylated forms were calculated after normalization to the total protein input. **B**. Inhibition of TRAF6-mediated NF-κB activation by WDR23. 293T cells were transfected with Flag-TRAF6 (0.5 μg), Xp-WDR23 (0.15–2.0 μg) and the NF-κB-Luc reporter (0.25 μg). At 24 h posttransfection, NF-κB activity was measured using a luciferase assay. The total protein in the cell lysates was used for normalization. The levels of Flag-TRAF6 and Xp-WDR23 expression were detected by anti-Flag and anti-Xp immunoblotting, respectively. β-Actin was used as the loading control. **C**. Inhibition of TRAF6-mediated AP-1 activation by WDR23. 293T cells were transfected with Flag-TRAF6 (0.5 μg), Xp-WDR23 (0.15–2.0 μg) or the AP-1-Luc reporter (0.25 μg). The data are shown as the means ± SD. ^*^*P*<0.05. ^**^*P*<0.01. ^***^*P*<0.001. n.s., not significant.

**Figure S5.** **WDR23 expression decreases TRAF6 stability.** **A**. Reduction in TRAF6 expression by WDR23. 293T cells were transfected with Xp-WDR23 (0.15–2 μg) and Flag-TRAF6 (0.5 μg). After 24 h, the cell lysates were analyzed by immunoblotting with anti-Flag and anti-Xp antibodies (top panel). β-Actin was used as the loading control. The relative levels of Flag-TRAF6 were normalized with β-actin (bottom panel). **B**. Reduction of endogenous TRAF6 by WDR23. 293T cells were transfected with increasing amounts of Xp-WDR23 (0.15–2 μg), and endogenous TRAF6 (TRAF6_endo_) levels were assessed by immunoblotting with an anti-TRAF6 antibody (top panel). The relative levels of endogenous TRAF6 were normalized with β-actin (bottom panel). **C**. The level of TRAF6_[1-129]_ by WDR23 expression. **D**. The level of TRAF6_[1-183]_ by WDR23 expression. **E**. The level of TRAF6_[1-300]_ by WDR23 expression. **F**. The level of TRAF6_[1-355]_ by WDR23 expression. **G**. The level of TRAF6_[356-530]_ by WDR23 expression. **H**. The levels of TRAF6 and TRAF6_[1-355]_ by WDR23 expression. **I**. The levels of TRAF6 and TRAF6_[356-530]_ by WDR23 expression. **J**. Decreased TRAF6 stability in the presence of WDR23. 293T cells were transfected with Xp-WDR23 (1.0 μg) and Flag-TRAF6 (1.0 μg). After 24 h, the cells were treated with cycloheximide (CHX, 50 μg/ml) for the indicated times. TRAF6 stability was analyzed by immunoblotting with anti-Flag antibodies (top panel). The relative levels of Flag-TRAF6 were normalized with β-actin (bottom panel). **K**. The stability of TRAF6_[1-129]_ by WDR23 expression. **L**. The stability of TRAF6_[1-300]_ by WDR23 expression. **M**. The stability of TRAF6_[1-355]_ by WDR23 expression. **N**. The stability of TRAF6_[356-530]_ by WDR23 expression. **O.** Reduction in TRAF6 levels via the TRAF6-WDR23 interaction in 293T cells. 293T cells were transfected with either Xp-WDR23 (0.15–2 μg) or Xp-WDR23_Δ[154–245]_ (0.15–2 μg) along with Flag-TRAF6 (0.5 μg). TRAF6 stability was assessed by immunoblotting with anti-Flag antibodies (top panel). The relative levels of Flag-TRAF6 were normalized to those of β-actin (bottom panel). The data are shown as the means ± SD. ^*^*P*<0.05. ^**^*P*<0.01. ^***^*P*<0.001. n.s., not significant.

**Figure S6. The reduction in TRAF6 stability caused by WDR23 is not affected by variations in promoter activity regulating TRAF6 expression. A**. TRAF6 stability driven by the CMV promoter (pCMV) is decreased following forced expression of WDR23. 293T cells were transfected with Flag-TRAF6 (pCMV-Flag-TRAF6 (0.5 μg)) and Xp-WDR23 (0.15-2.0 μg). After 24 h, the cell lysates were analyzed by immunoblotting with antibodies against Flag and Xp. β-Actin was used as the loading control. The specific bands are indicated by arrows. **B**. TRAF6 stability driven by the EF1α promoter (pEF1α) is decreased following forced expression of WDR23. 293T cells were transfected with Flag-TRAF6 (pEF1α-Flag-TRAF6 (0.5 μg)) and Xp-WDR23 (0.6-2.0 μg). The relative level of TRAF6 was normalized with β-actin.

**Figure S7. Among TRAF family members, the level of TRAF6 is selectively decreased in the presence of WDR23.** 293T cells were cotransfected with Flag-TRAFs (1.0 μg) and Xp-WDR23 (0.1-1.0 μg). After 24 h, the cell lysates were analyzed by immunoblotting with antibodies against Flag and Xp. β-Actin was used as the loading control. The specific bands are indicated by arrows.

**Figure S8. The co-localization between TRAF6 and WDR23 decreases TRAF6 protein levels in a time-dependent manner. A**. Representative imunofluorescence analysis of TRAF6 in MEFs. MEFs were transiently transfected with either Flag-WDR23 or the TRAF6-nonbinding mutant Flag-WDR23_Δ[154-245]_. The transfected cells were immunostained at 24 and 48 h post-transfection using an anti-Flag antibody followed by a FITC-conjugated secondary antibody to detect WDR23 (green), and an anti-TRAF6 Alexa Fluor 633-conjugated antibody to detect endogenous TRAF6 (magenta). The subcellular localization of TRAF6 and WDR23 was visualized by confocal microscopy (magnification, ×800). The fluorescence intensity profiles of TRAF6 and WDR23 were analyzed by line scan analysis (right panel). The nuclei were visualized by DAPI staining (blue). Scale bar: 10 μm. **B**. Representative imunofluorescence analysis of TRAF6 in BMMs. BMMs were infected with retroviral vectors encoding either Flag-WDR23 or Flag-WDR23_Δ[154-245]_. Immunostaining was performed at 24 and 48 h post-infection.

**Figure S9. The level of TRAF6 mRNA is not affected by WDR23 expression.** **A**. The level of TRAF6 mRNA is not affected by forced expression of WDR23. BMMs transduced with retroviral supernatants harboring Flag-WDR23 expression cassettes were selected with puromycin (2 μg/ml) in the presence of M-CSF (150 ng/ml) for 2 d. Puromycin-resistant Flag-WDR23-expressing BMMs were differentiated into BMOCs with M-CSF (50 ng/ml) and RANKL (200 ng/ml) for 4 d. The level of TRAF6 mRNA was analyzed by real-time PCR. **B**. The level of TRAF6 mRNA is not affected by WDR23^KD^. Puromycin-resistant WDR23^KD^-BMMs were differentiated into BMOCs with M-CSF (50 ng/ml) and RANKL (200 ng/ml) for 4 d. The level of TRAF6 mRNA was analyzed by real-time PCR. Mock, empty vector control. **C**. The level of TRAF6 mRNA is not different between WDR23^+/^*^+^-*BMMs and WDR23^-/-^-BMMs. WDR23^+/+^- and WDR23^-/-^-BMMs were differentiated into BMOCs with M-CSF (50 ng/ml) and RANKL (200 ng/ml) for 4 d. TRAF6 mRNA levels were analyzed by real-time PCR. The data are shown as the means ± SD. n.s., not significant.

**Figure S10. Optimization of inhibitor treatment concentrations and time points in 293T cells.** **A**. 293T cells co-expressing Flag-TRAF6 or Xp-WDR23 were treated with a ubiquitin‒proteasome inhibitor (MG132 or MG115) in a dose-dependent manner for 4 h. Cell lysates were analyzed by immunoblotting with antibodies against Flag and Xp, respectively. β-Actin was used as the loading control. **B-D**. 293T cells co-expressing Flag-TRAF6 and Xp-WDR23 were treated with autophagy inhibitors (3-MA, Wort or Baf A1) in a dose- and time-dependent manner. The cell lysates were analyzed by immunoblotting with antibodies against LC3B and Beclin1.

**Figure S11.** **WDR23 deficiency enhances RANKL-induced osteoclastogenesis. A**. Schematic diagram illustrating the generation of WDR23-deficient (WDR23^-/-^) mice using CRISPR-Cas9 targeting. The WDR23 genomic locus is shown with exons indicated by gray boxes and labeled with numbers. The transcription initiation site (black arrow), start codon (ATG) and stop codon (TAG) are marked. The CRISPR-Cas9 system targeted a cleavage site (CS with red arrow) at amino acid 18 on exon 2, leading to an aberrant sequence and premature stop (*), as shown in red. The exon 2 sequence (1-51 residues) is shown in black. **B**. Genomic PCR analysis of WDR23^-/-^ mice. Wild-type PCR bands (+/+), knockout bands (-/-) and markers (M) are displayed. **C**. Increased osteoclastogenesis in WDR23^-/-^-BMMs. BMMs from WDR23^+/+^ and WDR23^-/-^ mice were differentiated into BMOCs with M-CSF (50 ng/ml) and RANKL (200 ng/ml) for 4 d. Osteoclastogenesis was assessed by TRAP staining (top panels; original magnification ×50) and quantified by a TRAP activity assay (bottom left panel) and counting TRAP-positive multinucleated OCs (TRAP^+^ MNCs (≥three nuclei; bottom right panel). **D**. Increased NFATc1 mRNA expression in WDR23^-/-^-BMMs during osteoclastogenesis. NFATc1 expression was analyzed by real-time PCR. **E**. Upregulation of osteoclastogenic markers in WDR23^-/-^-BMMs. The expression of osteoclastogenic markers was measured by real-time PCR. The data are shown as the means ± SD. ^*^*P*<0.05. ^**^*P*<0.01. ^***^*P*<0.001. n.s., not significant.

**Figure S12. Optimization of inhibitor treatment concentrations in BMMs.** **A**. Optimization of ubiquitin‒proteasome inhibitor treatment concentrations in BMMs. BMMs expressing Xp-WDR23 were treated with ubiquitin‒proteasome inhibitors (MG132 or MG115) in a dose-dependent manner for 8 h. Cell lysates were analyzed by immunoblotting with antibodies against TRAF6 and Xp, respectively. β-Actin was used as the loading control. **B‒D**. Optimization of autophagy inhibitor treatment concentrations in BMMs. BMMs expressing Xp-WDR23 were treated with autophagy inhibitors (3-MA, Wort or Baf A1) in a dose-dependent manner. The cell lysates were analyzed by immunoblotting with antibodies against TRAF6 and Xp. **E**. Endogenous TRAF6 levels in BMMs and BMOCs following inhibitor treatment. BMMs were differentiated into BMOCs for 4 d in the absence (-) or presence (+) of RANKL stimulation and then treated with ubiquitin‒proteasome inhibitors or autophagy inhibitors for 8 h. Endogenous TRAF6 protein levels were analyzed by immunoblotting with antibodies against TRAF6 (top panels). The relative levels of endogenous TRAF6 were quantified and normalized to β-actin (bottom panels). The data are shown as the means ± SD. ^*^*P*<0.05. n.s., not significant.

**Figure S13. Optimization of treatment concentrations and time points for autophagy inducers in BMMs.** BMMs were treated with autophagy inducers (**A**, torin1 or **B**, rapamycin) in a dose- and time-dependent manner. The cell lysates were analyzed by immunoblotting with antibodies against LC3B and Beclin1. β-Actin was used as the loading control.

**Figure S14.** **WDR23 facilitates the recruitment of TRAF6 to autophagosomal compartments.** BMMs co-expressing Flag-WDR23 or Flag-WDR23_Δ[154-245]_ along with LC3-RFP were treated with either 100 nM torin1 (**A**) or 50 μM rapamycin (**B**) for 8 h. Cells were immunostained with a rabbit anti-TRAF6 antibody (magenta) and a mouse anti-Flag antibody (green). LC3-RFP puncta marking autophagosomes were visualized in red, and nuclei were counterstained with DAPI (blue). The areas surrounded by a white box are shown in greater magnification. Co-localization of TRAF6 with LC3-positive puncta was assessed by fluorescence intensity line scan analysis (middle panel) and quantitatively analyzed (right panel). Magnification: ×800. Scale bar: 10 μm. The data are shown as the means ± SD. ^**^*P*<0.01.

**Figure S15. Impaired bone formation in WDR23^-/-^ mice.** **A**. *In vivo* dynamic bone formation analysis of femurs from 8-week-old WDR23^-/-^ mice and control littermates. Mineralizing surface per bone surface (MS/BS), mineral apposition rate (MAR) and bone formation rate per bone surface (BFR/BS) were quantified. Scale bar, 50 μm. **B**. Expression of WDR23 in primary OBs. Primary OBs were isolated from the calvaria of newborn wild-type and WDR23**^-/-^** mice. WDR23 expression levels were assessed by immunoblotting using anti-WDR23 antibodies following parathyroid hormone (PTH) stimulation. β-Actin was used as the loading control. **C**. *In vitro* bone formation analysis of primary OBs derived from WDR23^-/-^ mice. OB differentiation and function were evaluated by alkaline phosphatase (ALP) activity assays at day 6 and alizarin red S (ARS) staining at day 21. **D**. Expression of OB marker genes of primary OBs derived from WDR23^-/-^ mice. The expression levels of osteocalcin and osteopontin were analyzed by real-time PCR analysis. The data are shown as the means ± SD. ^*^*P*<0.05. ^**^*P*<0.01. ^***^*P*<0.001.

**Figure S16. WDR23-mediated TRAF6 degradation is not affected by mutations in the DxR consensus binding motif of WDR23.** **A.** Schematic diagram of the two DxR consensus binding motifs implicated in the interaction with the CUL4A-DDB1 E3 ubiquitin ligase complex. The DxR motifs located within WD40 repeat domains 4 and 5 of WDR23 are highlighted. The mutated residues (substitution of arginine (R) with histidine (H)) in the DxR motifs are indicated at the bottom (red). **B.** Mutations in the DxR motif of WDR23 do not affect TRAF6 degradation. 293T cells were transfected with Xp-TRAF6 (1.0 μg) and Flag-WDR23 or WDR23-DxR mutants (0.1-1.0 μg). After 24 h, the cell lysates were analyzed by immunoblotting with antibodies against Flag and Xp. β-Actin was used as the loading control. The specific bands are indicated by arrows.

**Figure S17. The interaction between TRAF6 and WDR23 is not altered by mutation of the putative TRAF6-binding consensus motif in WDR23.** **A.** Schematic diagram illustrating the putative TRAF6-binding consensus motif in WDR23. The motif (PxExxZ, where Z represents either an acidic or aromatic residue) located within the WD40 repeat domain 3 (residues 261–266) is depicted. The mutated residues in the putative TRAF6-binding consensus motif (WDR23_P261A, E263A_) are indicated at the bottom. **B.** The WDR23_P261A,E263A_ mutant retains interaction with TRAF6. 293T cells were transfected with GST-TRAF6 (1.0 μg) and Xp-WDR23 or its WDR23_P261A,E263A_ mutant (1.0 μg). WDR23 binding to GST-TRAF6 was detected by anti-Xp immunoblotting (IB) (top panel). The levels of GST-TRAF6 determined by GST bead pulldown (PD) and Xp-WDR23 were analyzed by anti-GST IB (middle panel) and anti-Xp IB (bottom panel), respectively.
